# Chemicolome and Metabolome Profiling of Xieriga-4 Decoction, A Traditional Mongolian Medicine, Using the UPLC-QTOF/MS Approach

**DOI:** 10.1155/2022/8197364

**Published:** 2022-11-16

**Authors:** Yuanyuan Ma, Ruiting Ma, ZhiGui, QiLa Sa, Na Zhao, Wurigumala, Burentegusi, Zhigang Guo, Tumenwuliji

**Affiliations:** ^1^Inner Mongolia Medical University, Inner Mongolia, Hohhot 010010, China; ^2^Affiliated Hospital of Inner Mongolia Medical University, Inner Mongolia, Hohhot 010010, China; ^3^Life Sciences Department, Nanjing Normal University, Nanjing 210026, China; ^4^Clinical Lab Department, Inner Mongolia Autonomous Region Mental Health Center, Hohhot 010010, China

## Abstract

**Background:**

Xieriga-4 decoction (XRG-4) is a classic prescription Mongolian medicine that has potent diuretic and anti-inflammatory activities. However, its functional components remain unknown.

**Purpose:**

This study aimed to identify the chemical components in XRG-4 and its metabolome in vivo.

**Methods:**

An ultra-performance liquid chromatography coupled with a quadrupole time-of-flight tandem mass spectrometry based approach was proposed to systematically profile the chemicolome and metabolome of XRG-4.

**Result:**

A total of 106 constituents were identified in XRG-4. Eighty-nine components were identified in biological samples, including 78 in urine (24 prototypes and 54 metabolites), 26 in feces (19 prototypes and 7 metabolites), and 9 in plasma (5 prototypes and 4 metabolites). In other tissues, only a few compounds, including alkaloids and iridoids, were detected.

**Conclusion:**

This comprehensive investigation of the chemical and metabolic profiles of XRG-4 provides a scientific foundation for its quality control and administration of clinically-safe medication.

## 1. Introduction

Xieriga-4 decoction (XRG-4) is a classical Mongolian medicine prescription, which comprises four plants, including phellodendron chinensis cortex (PCC), tribuli fructus (TF), curcumae longae rhizoma (CLR), and gardenia fructus (GF). It is described in the Mongolian Medicine Volume of the Drug Standard of the Ministry of Public Health of the People's Republic of China (Commission, 1998), becoming a national registered standard preparation. XRG-4 has various pharmacological activities, such as diuresis and detumescence, renal protection, anti-inflammation, labor pain, and bacteriostasis [1, 2]. It is widely adopted in Mongolian medicine to treat urinary infectious diseases such as nephritis, cystitis, benign prostatic hyperplasia, and urinary tract infection. [3, 4]. After oral administration, components of XRG-4 are absorbed by the gastrointestinal tract to be further distributed, metabolized, and excreted. Despite their advantages in multiple-target and synergistic effects of Mongolian medicine, the specific in vivo targets of active ingredients remain unclear. Therefore, the pharmacodynamic bioactive components and the further mechanism are worth exploring.

Mass spectrometry is a novel, comprehensive method for rapid chemicolome analysis and has unique advantages in metabolism studies of traditional Chinese medicine (TCM) [5, 6]. Ultra-performance liquid chromatography coupled with Quadrupole time-of-flight tandem mass spectrometry (UPLC-QTOF/MS) has the characteristics of high sensitivity, mass accuracy, resolution, and wide scanning range. Analysis of the relative molecular mass, elemental composition, and fragmentation information of compounds can lead to the identification of various complex components and chemical structures of TCM to provide further technical support for the modern study of traditional Chinese medicine [7, 8].

In this research, the UPLC-QTOF/MS technique was used to identify and characterize the compounds in XRG-4. First, the components in XRG-4 were identified by UPLC-QTOF-MS and by using information from the in-house database. Next, the absorbed prototypes and their phase-I and phase-II metabolites were characterized, and their in vivo distribution was investigated. Finally, a network was built to reveal the relationship between metabolites and prototypes. Our research provides a scientific basis for further investigating pharmacological bioactive components and quality markers in XRG-4.

## 2. Material and Methods

### 2.1. Reagents and Medicinal Materials

XRG-4 was prepared by the Pharmaceutical Department, Inner Mongolia Mongolian Medicine Co. Ltd. Sixteen reference standards were purchased from Chengdu Pulse Biological Technology Co, Ltd. The purity of each compound was more than 98%, as determined by the UPLC analysis. Palmatine hydrochloride (China National Institute for Food and Drug Control; Batch No. 110732–201108); Compound Phellodendron Chinense Liquid (Shandong Hanfang Pharmaceutical Co. Ltd; Batch No. 20170412). Methanol, acetonitrile, and formic acid were all purchased from Thermo Fisher Technology Co. Ltd.

### 2.2. Preparation of XRG-4

0.2 g XRG-4 was ultrasound three times for 30 min in a conical bottle with 20 mL of 70% methanol, and the supernatant was centrifuged at 13000 rpm for 10 min. 400 *μ*L supernatant was transferred into a fresh tube and dried under nitrogen gas. The dried supernatant was redissolved with 400 *μ*L of 50% acetonitrile and centrifuged at 13000 rpm for 10 min. The supernatant was transferred into a new tube, and a 2.0 *μ*L aliquot was injected for UPLC-QTOF-MS.

### 2.3. Animal Experiments

Male Wistar rats (weight 150–200 g) were purchased from Jiangsu Ji Cui Yao Kang Biotechnology Co., Ltd. Animals were housed at 23 ± 2°C with 12 h light/dark cycle and had free access to a standard diet and water. A total of 12 rats were randomly categorized into two groups, six each for the control group and the administration group. After 3 days of acclimatization, the rats that were fasted for 16 h before dosing were administered an oral dose of 1.5 g/kg of XRG-4. All procedures were performed as per the guidance of the Provisions and General Recommendation of the Chinese Experimental Animals Administration Legislation.

### 2.4. Sample Collection and Preparation

*Plasma*: Blood samples were collected from the orbital venous plexus of rats at 5, 15, 30, 60, 120, 240, 360, 480, and 600 min post-administration. Nearly 200 *μ*L of plasma was mixed with 600 *μ*L of acetonitrile (containing 0.2% formic acid), and the mixture was vortexed for 2 min. Then, the samples were centrifuged at 13000 r/min (4°C) for 10 min, and 400 *μ*L of supernatant was dried under nitrogen gas and redissolved in 100 µL of acetonitrile (50%). The residue was reconstituted in 100 µL of acetonitrile-water (v/v, 1:1), and the obtained supernatant was used for UPLC-QTOF-MS analysis after centrifugation at 13000 rpm for 10 min.

Feces: Fecal samples were collected at 600 min pre- and post-administration and stored at -80°C before pretreatment. Feces were weighed and nearly 300 mg of the sample was mixed with 1 mL of methanol and magnetic beads. The mixture was ground at 40 Hz six times with a 5 s interval, with a total running time of 80 s. After grinding, the samples were centrifuged at 13000 r/min (4°C) for 10 min. Nearly 200 *μ*L of supernatant was obtained and dried under nitrogen gas; the residue was redissolved in 200 *μ*L of 50% acetonitrile. After centrifugation at 13,000 rpm for 10 min, a 2.0 *μ*L aliquot of the supernatant was obtained for UPLC-QTOF-MS analysis.

Urine: Urine samples were collected at 600 min pre- and post-administration and stored at -80°C. The urine was freeze-thawed and centrifuged at 4000 r/min for 10 min, and 1 mL of supernatant was loaded onto an activated C18 SPE column (waters). The loaded column was washed with 1 mL of ultrapure water, eluted with 1 mL of methanol and collected and centrifuged at 13000 rpm for 10 min at 4°C. Nearly 400 *μ*L of supernatant was transferred and dried under nitrogen gas. The residue was redissolved in 400 *μ*L of acetonitrile (50%), centrifuged at 13000 rpm for 10 min at 4°C, and a 2.0 *μ*L of aliquot was injected into UPLC-QTOF-MS.

*Tissue*: The rats were sacrificed 600 min post-dosing. Then the heart, liver, lungs, spleen, kidneys, and brain were harvested and homogenized. The tissue samples were weighed (300 mg) and mixed with 1 mL of methanol and magnetic beads in a 2 mL centrifuge tube. The mixture was ground six times at 40 Hz with a 5 s interval for a total run time of 80 s. After grinding, the samples were centrifuged at 13000 r/min (4°C) for 10 min. The supernatant (200 *μ*L) was dried under nitrogen gas, and the residue was redissolved in 200 *μ*L of acetonitrile (50%). This was centrifuged at 13,000 rpm for 10 min, and a 2.0 *μ*L of aliquot of the supernatant was used for UPLC/Q-TOF MS analysis.

### 2.5. Analysis Condition for Ultra-performance Liquid Chromatography Coupled with Quadrupole Time-of-Flight Tandem Mass Spectrometry

The supernatant (2.0 *μ*L) obtained as described in the previous step was used for chromatographic separation on an Exion LC system (AB Sciex, Foster City, CA, USA). The Waters Acquity HSS T3 column (2.1 × 150 mm; 1.7 *μ*m) was used at 35°C. For the mobile phase, eluent A (water with 0.1% formic acid, v/v) and eluent B (acetonitrile) were used. The linear elution gradient program was optimized as follows: 0–5 min from 3% to 8% B, 5–11 min from 8% to 30% B, 11–20 min from 30% to 80% B, 20–21 min from 80% to 95% B, 21–29 min at 95% B, then back to the initial ratio of 3% B and maintained for additional 4.5 min for re-equilibration.

## 3. Results

### 3.1. Identification of Chemical Components in XRG-4

**Figure 1:**
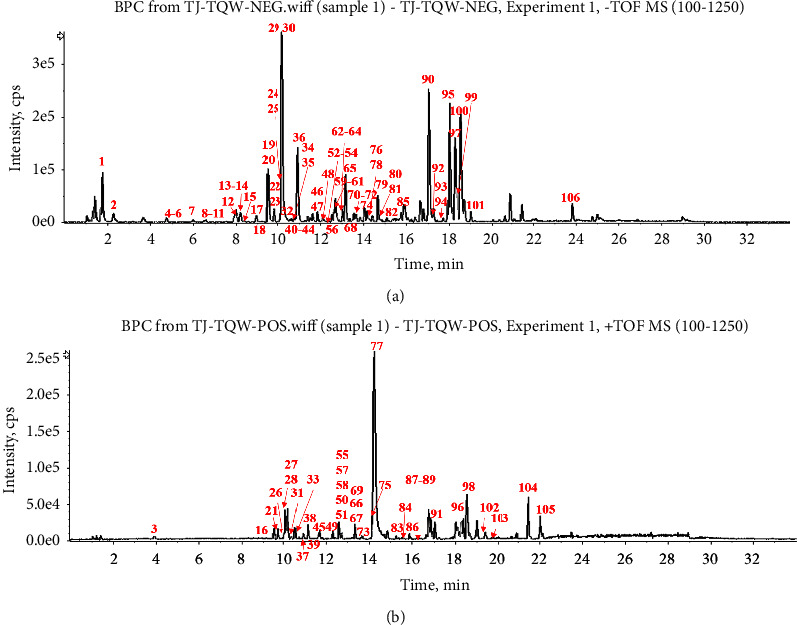
The base peak chromatograms of XRG-4 (a) negative ion mode; (b) positive ion mode.

**Figure 2:**
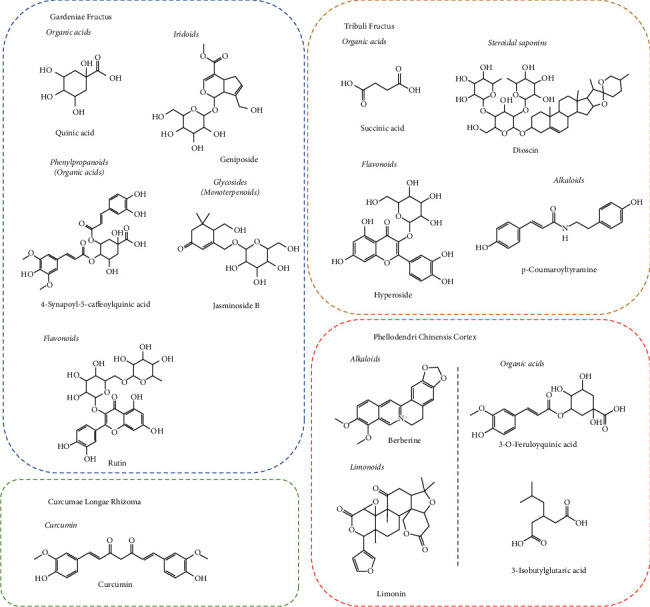
The main chemical structures of components identified in XRG-4.

The base peak chromatograms in positive and negative modes of XRG-4 in UPLC-QTOF-MS analysis are shown in Figure 1. A total of 106 compounds in XRG-4 were identified. Among them, 32 compounds exist in PCC, 21 in TF, 16 in GLR, and 47 in GF. As shown in Table S1. The representative components of each plant are shown in Figure 2. The main characteristic components in PC were alkaloids, limonin, and phenylpropanoids (organic acid); TF contains more saponins with a molecular weight >900 (Da), some alkaloids, and a few flavonoids. The main characteristic components of GLR were the curcumins, the phenylpropanoid derivatives. A variety of chemical types were found in GF, such as triterpenoids, phenylpropanoid derivatives, flavonoids, and iridoids.

### 3.2. Mechanism of Fragmentation of Representative Structures

**Figure 3:**
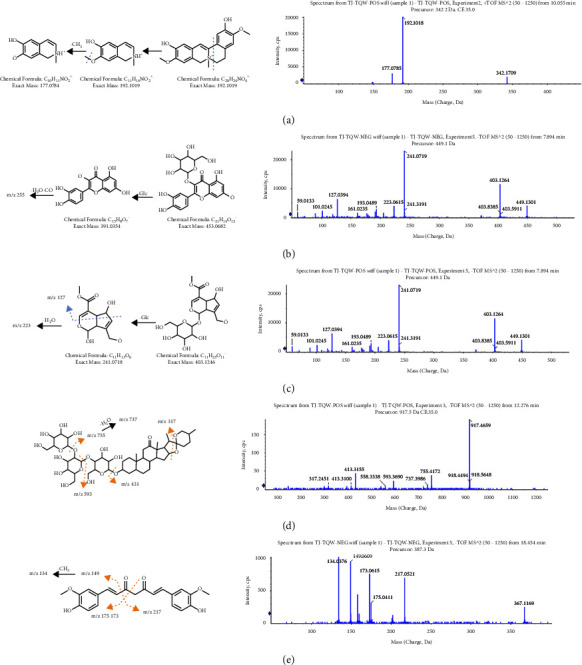
The proposed fragmentation pathway of characteristic components in XRG-4. (a) Phellodendrine; (b) hyperoside; (c) feretoside; (d) hecogenin 3-O-*β*-glucopyranosyl (1⟶2)-*β*-glucopyranosy l (1⟶4)-galactopyranoside; and (e) curcumin.

The proposed fragmentation patterns of typical compounds in XRG-4 are shown in Figure 3.

#### 3.2.1. Phellodendron Chinensis Cortex

PCC has various pharmacological actions, such as anti-inflammatory, anti-tumor, and hypoglycemic. The most characteristic components found in PCC were alkaloids, which generally have a good response in the positive ion mode, considering the fragmentation patterns of *Phellodendrine* as an example. As shown in Figure 3(a), the fragmentation of heterocyclic rings produced ions at *m/z* 192, and subsequent demethylation (△*m* = 15) resulted in product ions at *m/z* 177. A total of 32 active components were identified in PCC, including 18 alkaloids such as candicine (P3), phellodendrine oxide (P26), N-methylhigenamine-7-glucopyranoside (P27), and berberine (P77) (Table S2).

#### 3.2.2. Gardenia Fructus

GF shows multiple biological activities in clinical practice, such as anti-oxidant properties, hypoglycemic effect, inhibition of inflammation, anti-depression activity, and improvement in sleeping quality [9]. The flavonoids and iridoids detected in this study were mainly sourced from GF. Hyperoside, a flavonoid, produced characteristic flavonoid aglycones ions (*m/z* 301, *m/z* 300) after deglycosylation, and subsequent loss of–CO and H2O produced ions at m/*z* 255 (Figures 3(b) and 3(c)). The main fragments (*m/z* 241) of feretoside, the iridoids, were obtained from deglycosylation and dehydration (*m/z* 223), aglycones cracking (*m/z* 127), and glycosylated cracking (*m/z* 161, *m/z* 101, *m/z* 59), which produced a series of fragment ions with lower responses. Finally, a total of 47 active components were identified in GF, including 21 flavonoids and iridoids, such as gardoside (P4), diacetyl asperulosidic acid (P5), geniposidic acid (P6), and shazhiside or its isomer (P8), the details of which are shown in Table S2.

#### 3.2.3. Tribuli Fructus

TF has anti-infective and diuretic activities and has been used to treat hypertension and edema. TF contains steroidal saponins, alkaloids, and flavonoids [10]. The fragments of hecogenin 3-O-*β*-glucopyranosyl (1⟶2)-*β*- glucopyranosyl (1⟶4) galactopyranoside were produced at m/*z* 755, m/*z* 593, *m/z* 431 by glycosyl losses, and m/*z* 317 by steroidal ring cracking, respectively. The typical fragmentation pathways of TF are drawn in Figure 3(d). A total of 21 components were identified in TF, including eight steroidal saponins, such as tribufuroside I (P40), tribulosaponin A (P52), hecogenin3-O-*β*-glucopyranosyl (1⟶2)-*β*-glucopyranosyl (1⟶4) galactopyranoside (P55), and oxy-berberine (P58) (for details, see Table S2).

#### 3.2.4. Longue Rhizoma

CLR has been reported to exhibit anti-oxidant, anti-inflammatory, hepatoprotective, anti-atherosclerotic, and anti-diabetic properties [11]. Curcumins are the characteristic components in CLR, which is also a phenylpropanoid derivative and a member of the diketone family. The fragments detected in its MS/MS spectra (*m/z* 217, *m/z* 175, *m/z* 173, *m/z* 149, and *m/z* 134) were generated by alkane chain breaks (Figure 3(e)). In our study, a total of 16 components were identified in GLR, including seven curcumins, such as tetrahydroxidemethoxycurcumin (P93), dihydrobisdemethoxycurcumin (P94), bisdemethoxycurcumin (p95), dihydrodemethoxycurcumin (p96), dihydrocurcumin (P99), and curcumin (P100), and the details are shown in Table S2.

### 3.3. Identification of Prototypes and Metabolites of XRG-4 in Rat Biological Samples

Some compounds (prototypes and some metabolic types), after being administered, are absorbed in the gastrointestinal tract and then sent to the liver through the portal vein for further metabolism. The circulation system allows for the systemic distribution of the components in various tissues and organs. Finally, the chemicals are excreted in the urine by the kidneys, and other components that are not absorbed by the gastrointestinal tract are excreted in the feces.

**Figure 4:**
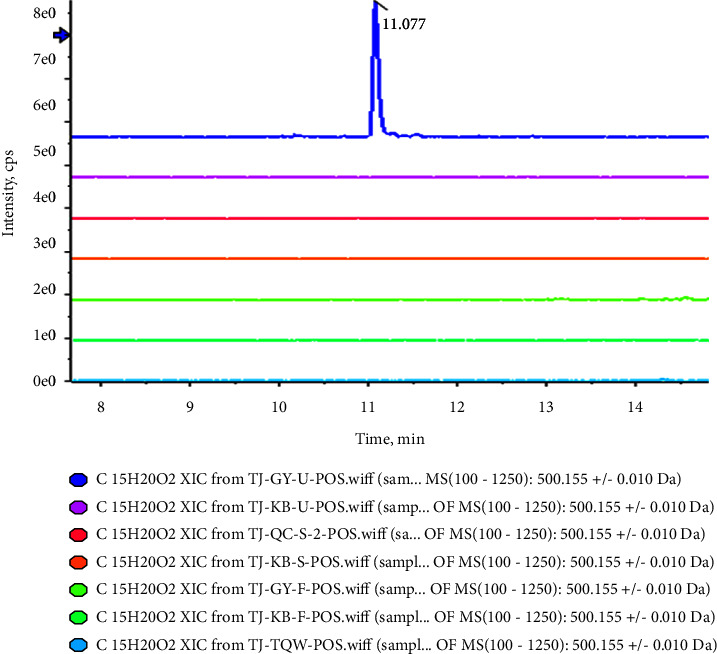
Overlayed extraction ion flow diagram of C_25_H_26_NO_10_ (M1) in urine, blank urine, plasma, blank plasma, feces, and blank feces.

**Figure 5:**
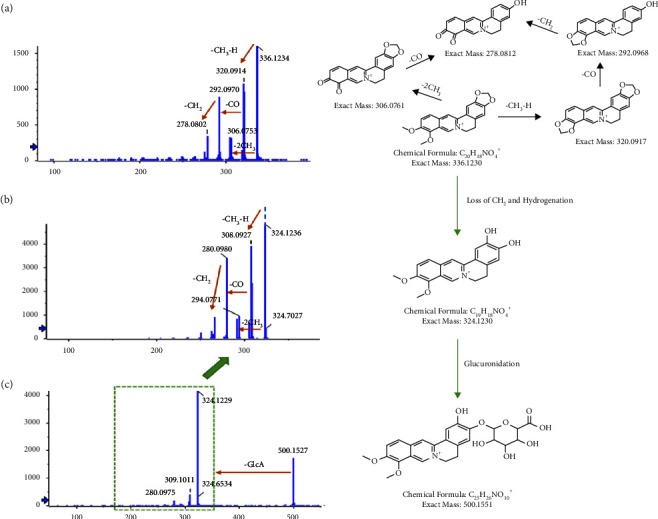
Metabolite identification and matching of P77 (berberin).

**Figure 6:**
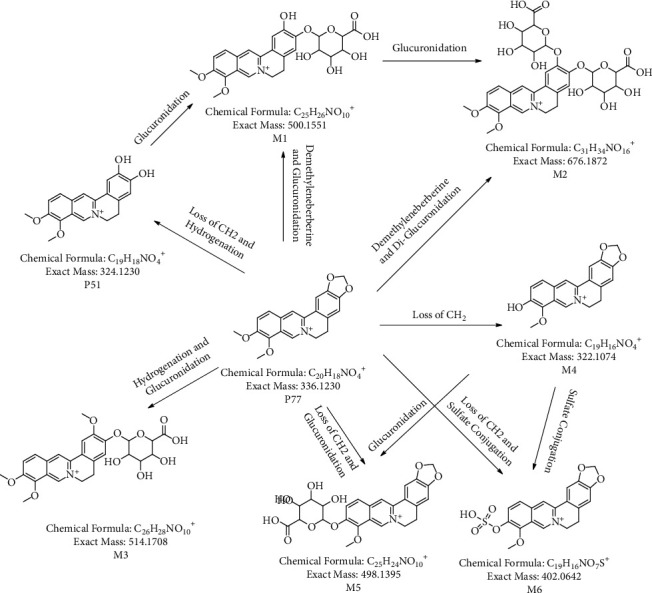
The network association diagram of P77 and its metabolic components.

In the current study, accurate mass measurement was performed to characterize the XRG-4 compounds, their retention times, and ms/ms fragmentation behaviors; some compounds were identified by corresponding reference standards. Prototypical components were extracted from plasma, urine, and feces through the rules of phase I and phase II metabolism and showed similar secondary mass spectrometry profiles. The base peak chromatograms of XRG-4 in plasma, urine, and feces in both negative and positive modes are presented in Figures S1-S3. As a result, the prototype components could be quickly screened from the metabolite substance, automatically matched with the prototype components, and assisted in the identification and annotation of metabolites. Taking P77 (berberine)-C25H26NO10 (M1; demethyleneberberine and glucuronidation) as an example, a significant response of metabolites was observed only in the urine after drug administration (Figure 4). This indicates that M1 may be a metabolite generated by absorption and metabolism post-drug administration and finally discharged from the body through urine. The secondary mass spectrometry and fragmentation mode of P77 (berberine) (Figure 5(a)) shows a series of fragments produced by -CH3-H (−16 Da), −2CH3 (−30 Da), -Co (−28 Da), and -CH2 (−14 Da). Figure 5(b) shows the secondary map of P51 (demethyleneberberine), which is also one of the metabolites (produced by loss of CH2 and hydrogenation) of berberine and exhibits the same fragmentation mode as berberine. Figure 5(c) presents the secondary mass spectrometry map of M1 (C25H26NO10, demethyleneberberine, and glucuronidation). There is a neutral loss of glucoside (−176 Da), and the parent nucleus is highly similar to demethyleneberberine; hence, M1 was classified as one of the metabolites of berberine. According to this principle, seven metabolites matched with the prototype berberine, and the structure and biotransformation correlation diagram are presented in Figure 6.

**Figure 7:**
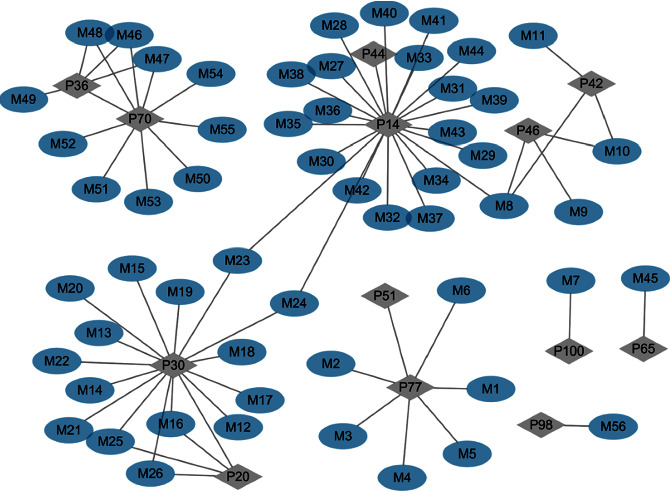
The network association diagram of 11 representative structures.

A total of 11 representative structures, berberine (P77, alkaloids), curcumin (P100, curcumin), isoquercetin/hyperoside (P46, flavonoids), rutin (P42, flavonoids), geniposide (P30, iridoids), genipin 1-gentiobioside (P20, iridoids), jsminoside B/F (P14, 2-ISObutylglutaric acid (P65, organic acids), 3-O-feruloyl quinic acid (P36, 2-O-feruloyl quinic acid, organic acids), 4-sinapoyl-5-caffeoylquinic acid (P70, organic acids), and dioscin (P98, nasal saponins), were selected for metabolite identification and prototype-metabolic matching. A total of 56 metabolic components are finally matched, and the associated network between related prototypes and metabolic compounds is prepared, as shown in Figure 7 and Table S2.

## 4. Discussion

XRG-4 is a classic Mongolian medicine prescription. In 1998, it was included in the Pharmaceutical Standards of the Ministry of Health of the People's Republic of China-Mongolian Medicine. Presently, XRG-4 has diuretic [12, 13] and anti-inflammatory [14–16] effects, but its potential hypoglycemic activities and bioactive components have not been reported [17, 18].

Eleven representative structures were selected from the experiment. These natural compounds have a potential role in preventing or controlling diabetes mellitus. The underlying mechanism of the antidiabetic effects of these compounds include improvement in insulin secretion, decrease in insulin resistance, enhanced glycogen synthesis in the liver, and antioxidant and anti-inflammatory activities [19, 20]. Berberine, an alkaloid, is the main component of PCC. It effectively reduces fasting plasma glucose, postprandial blood glucose, and glycosylated hemoglobin by participating in insulin resistance, anti-inflammatory, antioxidation, regulating lipid metabolism disorders and intestinal flora, and other methods. Berberine is mainly used in the treatment of type 2 diabetes, obesity, and metabolic diseases [21–27]. We detected berberine in plasma, feces, and biological samples, indicating that the prototype and metabolites of berberine are involved in the hypoglycemic mechanism. It is the main bioactive component of XRG-4 in hypoglycemia. Curcumin is a characteristic component of CLR and exhibits anti-inflammatory, antioxidation, antitumor, and immunoregulatory properties. Moreover, it improves insulin resistance, obesity, and other FFA-related diseases [11, 28–31]. In this study, isoquercetin and rutin were also screened as representative compounds. Agarwal [32] reported the actions of isoquercetin and rutin, including antihyperglycemia and their effects on diabetic complications. Geniposide is a new type of iridoid glycoside, which is the main active ingredient of gardenia. Recent studies have found a variety of pharmacological and biological activities of Geniposide, including liver protection, antiosteoporosis, antitumor, antidiabetes, neuroprotection, and so on. In summary, the 11 key compounds detected in this experiment have certain efficacy and potential in addressing diabetes and its complications.

A variety of small molecular compounds were identified by UPLC-QTOF-MS. The bioactive components and metabolites of XRG-4 in plasma, urine, feces, and tissue samples in vivo, the metabolic pathways of XRG-4, and the prototype compounds were matched. In addition, the absorption, distribution, metabolism, and excretion pathways of XRG-4 were also determined in vivo. We could also summarize the distribution of prototype and structural representative metabolites in biological samples. Eighty-nine compounds were detected in biological samples and 78 in urine, including 24 prototypes and 54 metabolites. 26 compounds were detected in feces, including 19 prototypes and 7 metabolites, and 9 compounds were detected in plasma, including 5 prototypes and 4 metabolites. The distribution of bioactive components in vivo is shown in Table S3. In the metabolic process, urine is created by kidney filtration and reabsorption of blood through the glomerulus. Therefore, compounds excreted through the urine have undergone a systematic circulation system.

Only a few compounds, mainly alkaloids and iridoids, were detected in other tissues. Berberine was detected in all tissues and was probably the most widely distributed chemical component. Columbamine, palmatine, and jatrorrhizine were detected in the heart and kidney, and berberrubine was detected in the liver. Among the metabolites with tissue distribution, M1 is the metabolite of berberine, M16 and M17 are the metabolites of geniposide, and M43 is a metabolite of jasminoside B/F. These compounds may be worth further study for quality control and pharmacodynamic activities.

We detected only a few compounds in the plasma, possibly due to (1) the rapid excretion process of the chemicals and short residence time resulting in no detection in the blood; and (2) compounds combined with plasma protein at a higher rate, and after sample preparation, the low concentration in the plasma limits the detection of the object under test. In summary, the latest experimentation technology was used to discover the bioactive components of XRG-4 in vivo and in vitro, which helped to reveal the potential components of Mongolian medicine in vivo, further clarify its hypoglycemic activities and lay an experimental foundation for a clinical search of effective natural hypoglycemic drugs with few toxic and side effects.

## Data Availability

The raw data required to reproduce these findings cannot be shared here, as the data also form part of an ongoing study.

## Supplementary Materials

Supplementary Materials Supplementary information available: Tables S1-S3 and Figures S1-S3.
