# Supplementary material for: Chemicolome and Metabolome Profiling of Xieriga-4 Decoction, A Traditional Mongolian Medicine, Using the UPLC-QTOF/MS Approach
Source: Evid Based Complement Alternat Med. 2022 Nov 16;2022:8197364. doi: 10.1155/2022/8197364 (PMC9683986; doi:10.1155/2022/8197364)
Supplement: Supplementary Materials — Supplementary information available: Tables S1-S3 and Figures S1-S3. [file 8197364.f1.zip › supplementary meterial.pdf]

# Supplemental material

1 . Result

1.1 Identification of chemical components in XRG-4

The base peak chromatograms in positive and negative modes of XRG-4 in UPLC-QTOF-MS analysis are shown in Table S1. A total of 106 compounds in XRG-4 were identified. Among them, 32 compounds exist in PCC, 21 in TF, 16 in GLR, and 47 in GF.

1.2 Screening the XRG-4 absorbed in rats

1.2.1 A total of 11 representative structures, berberine (P77, alkaloids), curcumin (P100, curcumin), isoquercetin/hyperoside (P46, flavonoids), rutin (P42, flavonoids), geniposide (P30, iridoids), genipin 1-gentiobioside (P20, iridoids), jsminoside B/F (P14, 2-ISObutylglutaric acid (P65, organic acids), 3-O-feruloyl quinic acid (P36, 2-O-feruloyl quinic acid) organic acids), 4-sinapoyl-5-caffeoylquinic acid (P70, organic acids), dioscin (P98, nasal saponins), were selected for metabolite identification and prototype-metabolic matching. A total of 56 metabolic components were finally matched, and the associated network between related prototypes and metabolic compounds was prepared, as shown in Table S2.

1.2.2 A total of 89 compounds were detected in biological samples, 78 compounds were detected in urine, including 24 prototypes and 54 metabolites. A total of 26 compounds were detected in feces, including 19 prototypes and 7 metabolites. A total of 9 compounds were detected in plasma, including 5 prototypes and 4 metabolites. Distribution of substance asis in vivo were showed in Table S3

1.2.3 In the current study, the XRG-4 compounds were characterized by accurate mass measurement, retention time, MS fragmentation behaviors and certain reference standards. Phase I and phase II metabolites were both used to detected in plasma samples. Extracting prototypical components from plasma, urine, and feces, through the rule of phase I and phase II metabolism, with the similarity of secondary mass

spectrometry profiles. The negative and positive ions base peak chromatograms of XRG-4 in Plasma, urine, feces were showed in Figure S1-S3.

TableS1 Chemical composition characterization of XRG-4

| NO. | Compound Name | Formula | ESI- | | | | | | ESI+ | | | | | Class | Source |
|---|---|---|---|---|---|---|---|---|---|---|---|---|---|---|---|
| | | | **RT min** | **Ion Mode** | **Calc*m/z*** | ***m/z*** | **ppm** | **Fragment ions(*m/z*)** | **Ion Mode** | **Calc*m/z*** | ***m/z*** | **ppm** | **Fragment ions(*m/z*)** | | |
| P1 | Quinic acid | $C_7H_{12}O_6$ | 1.43 | [M-H]- | 191.0561 | 191.0566 | 2.56 | 173, 127, 93, 87, 85 | - | - | - | - | - | Organic acids | GF |
| P2 | Succinic acid | $C_4H_6O_4$ | 2.39 | [M-H]- | 117.0193 | 117.0196 | 2.31 | 99, 73 | - | - | - | - | - | Organic acids | T |
| P3 | Candicine | $C_{11}H_{18}NO^+$ | 3.89 | - | - | - | - | - | [M]+ | 180.1383 | 180.1385 | 1.11 | 121,77 | Alkaloids | PCC |
| P4 | Gardoside | $C_{16}H_{22}O_{10}$ | 5.01 | [M-H]- | 373.1140 | 373.1134 | -1.66 | 211, 193, 167, 149, 123 | - | - | - | - | - | Iridoids | GF |
| P5 | Deacetyl asperulosidic acid | $C_{16}H_{22}O_{11}$ | 5.07 | [M-H]- | 389.1089 | 389.1086 | -0.87 | 227, 209, 183, 183, 165, 147, 139 | - | - | - | - | - | Iridoids | GF |
| P6 | Geniposidic acid | $C_{16}H_{22}O_{10}$ | 5.75 | [M-H]- | 373.1140 | 373.1134 | -1.66 | 211, 193, 167, 149, 123 | - | - | - | - | - | Iridoids | GF |
| P7 | 2-Hydroxy-3-O-β-D-glucopyranosylbenzoic acid | $C_{13}H_{16}O_9$ | 5.87 | [M-H]- | 315.0722 | 315.0726 | 1.40 | 152, 108 | - | - | - | - | - | Phenyls | GF |

| | | | | | | | | | | | | | | | |
|---|---|---|---|---|---|---|---|---|---|---|---|---|---|---|---|
| P8 | Shazhiside or its isomer | $C_{16}H_{24}O_{11}$ | 5.99/6.58 | [M-H]- | 391.1246 | 391.1244 | -0.49 | 229, 211, 193, 185, 167, 149, 121, 109 | - | - | - | - | - | Iridoids | GF |
| P9 | 4-Methoxy benzonic acid-3-O-glucoside | $C_{14}H_{18}O_9$ | 6.7 | [M-H]- | 329.0878 | 329.0866 | -3.68 | 167, 152 | - | - | - | - | - | Phenyls | GF |
| P10 | Gentisic acid | $C_7H_6O_4$ | 6.92 | [M-H]- | 153.0193 | 153.0194 | 0.46 | 109, 108, 91, 81 | - | - | - | - | - | Benzoic acids | GF |
| P11 | Ixoroside | $C_{16}H_{24}O_9$ | 7.16 | [M+HCOO]- | 405.1402 | 405.1407 | 1.14 | 359, 197, 179, 117 | - | - | - | - | - | Iridoids | GF |
| P12 | Feretoside | $C_{17}H_{24}O_{11}$ | 7.97 | [M+HCOO]- | 449.1301 | 449.1295 | -1.25 | 403, 371, 241, 223, 193, 161, 127, 101, 89, 59 | - | - | - | - | - | Iridoids | GF |
| P13 | Neochlorogenic acid | $C_{16}H_{18}O_9$ | 8.08 | [M-H]- | 353.0878 | 353.0887 | 2.52 | 191, 179, 135 | - | - | - | - | - | Organic acids | PCC, GF |
| P14 | Jasminoside B/F | $C_{16}H_{26}O_8$ | 8.14 | [M-H]- | 345.1555 | 345.1562 | 2.06 | 179, 165, 119, 89, 59 | - | - | - | - | - | Glycosides | GF |
| P15 | Gardenoside | $C_{17}H_{24}O_{11}$ | 8.37 | [M+HCOO]- | 449.1301 | 449.1295 | -1.25 | 403, 371, 241, 223, 193, 161, 127, 101, 89, 59 | - | - | - | - | - | Iridoids | GF |

| | | | | | | | | | | | | | | | |
|---|---|---|---|---|---|---|---|---|---|---|---|---|---|---|---|
| P16 | Jasminoside D/G | $C_{16}H_{26}O_8$ | 8.92 | - | - | - | - | - | [M+H]+ | 347.1700 | 347.1710 | 2.77 | 185, 167, 137,109 | Glycosides | GF |
| P17 | 2-(P-hydroxy-phenyl)ethanol-1-O-beta-Glucoside | $C_{19}H_{28}O_{11}$ | 8.99 | [M-H]- | 431.1559 | 431.1558 | -0.21 | 299, 191, 149, 101, 89 | - | - | - | - | - | Phenyls | PCC |
| P18 | Mussaenosidic acid/Loganic acid | $C_{16}H_{24}O_{10}$ | 9.35 | [M-H]- | 375.1297 | 375.1288 | -2.32 | 213, 169, 151, 125, 89 | - | - | - | - | - | Iridoids | GF |
| P19 | Chlorogenic acid | $C_{16}H_{18}O_9$ | 9.45 | [M-H]- | 353.0878 | 353.0887 | 2.52 | 191, 179, 161, 127 | - | - | - | - | - | Organic acids | PCC, GF |
| P20 | Genipin 1-gentiobioside | $C_{23}H_{34}O_{15}$ | 9.45 | [M+HCOO]- | 595.1880 | 595.1891 | 1.90 | 549, 517, 225, 207, 179, 123, 101 | - | - | - | - | - | Iridoids | GF |
| P21 | Clausenamide | $C_{18}H_{19}NO_3$ | 9.65 | - | - | - | - | - | [M+H]+ | 298.1438 | 298.1444 | 2.11 | 283,254 | Alkaloids | PCC |
| P22 | Cryptochlorogenic acid | $C_{16}H_{18}O_9$ | 9.66 | [M-H]- | 353.0878 | 353.0887 | 2.52 | 191, 179, 135 | - | - | - | - | - | Organic acids | PCC, GF |
| P23 | 5-O-Feruloylquinic acid | $C_{17}H_{20}O_9$ | 9.74 | [M-H]- | 367.1035 | 367.1034 | -0.16 | 193, 191, 173, 149, 134 | - | - | - | - | - | Organic acids | PCC |
| P24 | Vanillic acid | $C_8H_8O_4$ | 9.81 | [M-H]- | 167.0350 | 167.0357 | 4.31 | 152, 123, 108 | - | - | - | - | - | Organic acids | T |
| P25 | 3/4/5-O-Sinapoylquinic acid | $C_{18}H_{22}O_{10}$ | 9.9 | [M-H]- | 397.1140 | 397.1122 | -4.58 | 223, 191, 164, 149 | - | - | - | - | - | Organic acids | GF |

| | | | | | | | | | | | | | | | |
|---|---|---|---|---|---|---|---|---|---|---|---|---|---|---|---|
| P26 | Phellodendrine oxide | $C_{20}H_{21}NO_5$ | 9.92 | - | - | - | - | - | [M+H]+ | 356.1493 | 356.1499 | 1.83 | 206,191 | Alkaloids | PCC |
| P27 | N-Methylhigenamine 7-glucopyranoside | $C_{23}H_{29}NO_8$ | 10.05 | - | - | - | - | - | [M+H]+ | 448.1966 | 448.1971 | 1.14 | 286, 255, 178, 107 | Alkaloids | PCC |
| P28 | Tetrahydrojatrorrhizine | $C_{20}H_{23}NO_4$ | 10.09 | - | - | - | - | - | [M+H]+ | 342.1700 | 342.1710 | 2.98 | 192,177 | Alkaloids | PCC |
| P29 | Caffeic acid | $C_9H_8O_4$ | 10.15 | [M-H]- | 179.0350 | 179.0347 | -1.56 | 135, 134, 108, 79 | - | - | - | - | - | Organic acids | GF |
| P30 | Geniposide | $C_{17}H_{24}O_{10}$ | 10.17 | [M+HCOO]- | 433.1352 | 433.1338 | -3.12 | 387, 225, 207, 147, 123, 101, 69 | - | - | - | - | - | Iridoids | GF |
| P31 | Tembetarine | $C_{20}H_{26}NO_4^+$ | 10.37 | - | - | - | - | - | [M]+ | 344.1856 | 344.1862 | 1.66 | 299, 286, 175, 137 | Alkaloids | PCC |
| P32 | Picrocrocinic acid | $C_{16}H_{26}O_8$ | 10.42 | [M-H]- | 345.1555 | 345.1555 | 0.03 | 179, 165, 119, 89, 59 | - | - | - | - | - | Glycosides | GF |
| P33 | Phellodendrine | $C_{20}H_{24}NO_4^+$ | 10.53 | - | - | - | - | - | [M]+ | 342.1700 | 342.1710 | 2.98 | 297, 282, 265, 237, 194, 191 | Alkaloids | PCC |
| P34 | Picrocrocin | $C_{16}H_{26}O_7$ | 10.73 | [M+HCOO]- | 375.1661 | 375.1651 | -2.56 | 179, 161, 119, 89 | - | - | - | - | - | Glycosides | GF |
| P35 | 6'-O-trans-Coumaroyl geniposidic acid/2'-O-trans-p- | $C_{25}H_{28}O_{12}$ | 10.75 | [M-H]- | 519.1508 | 519.1503 | -0.96 | 307, 211, 193, 167, 163, 145, 123 | - | - | - | - | - | Iridoids | GF |

| | | | | | | | | | | | | | | | |
|---|---|---|---|---|---|---|---|---|---|---|---|---|---|---|---|
| | Coumaroylgardoside | | | | | | | | | | | | | | |
| P36 | 3-O-Feruloylquinic acid | $C_{17}H_{20}O_9$ | 10.92 | [M-H]- | 367.1035 | 367.1034 | -0.16 | 193, 191, 173, 149, 134 | - | - | - | - | - | Organic acids | PCC |
| P37 | Quercetin 3,7-diglucoside/Quercetin 3-gentiobioside | $C_{27}H_{30}O_{17}$ | 10.95 | [M-H]- | 625.1410 | 625.1387 | -3.71 | 301 | [M+H]+ | 627.1556 | 627.1532 | -3.79 | 303 | Flavonoids | T |
| P38 | Lotusine | $C_{19}H_{23}NO_3$ | 11.14 | - | - | - | - | - | [M+H]+ | 314.1751 | 314.1745 | -1.81 | 269, 237, 143, 107 | Alkaloids | PCC |
| P39 | Jasminoside C | $C_{16}H_{24}O_7$ | 11.25 | - | - | - | - | - | [M+H]+ | 329.1595 | 329.1608 | 4.01 | 167, 149,111,98 | Glycosides | GF |
| P40 | Tribufuroside I | $C_{51}H_{84}O_{26}$ | 11.29 | [M-H]- | 1111.51781 | 1111.5173 | -0.46 | 949, 787 | - | - | - | - | - | Steroid saponins | T |
| P41 | 4-O-Feruloylquinic acid | $C_{17}H_{20}O_9$ | 11.43 | [M-H]- | 367.1035 | 367.1034 | -0.16 | 191, 173, 134, 93 | - | - | - | - | - | Organic acids | PCC |
| P42 | Rutin | $C_{27}H_{30}O_{16}$ | 11.52 | [M-H]- | 609.1461 | 609.1449 | -1.99 | 343, 301, 300, 285, 255, 217, 179, 151 | [M+H]+ | 611.1607 | 611.1623 | 2.68 | 465, 356, 303, 287, 147, 129, 85, 71 | Flavonoids | GF, T |
| P43 | Vanillin | $C_8H_8O_3$ | 11.53 | [M-H]- | 151.0401 | 151.0404 | 2.18 | 136, 108, 92 | [M+H]+ | 153.0546 | 153.0536 | -6.66 | 125, 111, 93, 65 | Phenyls | T |

| | | | | | | | | | | | | | | | |
|---|---|---|---|---|---|---|---|---|---|---|---|---|---|---|---|
| P44 | Jasminodiol | $C_{10}H_{16}O_3$ | 11.6 | [M-H]- | 183.10267 | 183.1032 | 2.89 | 139 | - | - | - | - | - | Terpene | GF |
| P45 | Menisperine | $C_{21}H_{26}NO_4^+$ | 11.68 | - | - | - | - | - | [M]+ | 356.1856 | 356.1857 | 0.20 | 311,296, 279,264,284,236, 219,191 | Alkaloids | PCC |
| P46 | Isoquercetin/Hyperoside | $C_{21}H_{20}O_{12}$ | 11.85 | [M-H]- | 463.0882 | 463.0876 | -1.30 | 301, 300, 271, 255, 179, 151 | - | - | - | - | - | Flavonoids | GF, T |
| P47 | 6'-O-trans-Sinapoyl gardoside | $C_{27}H_{32}O_{14}$ | 12.09 | [M-H]- | 579.1719 | 579.1716 | -0.57 | 385, 367, 325, 295, 265, 223, 205, 193, 123 | - | - | - | - | - | Iridoids | GF |
| P48 | Ferulic acid | $C_{10}H_{10}O_4$ | 12.12 | [M-H]- | 193.0506 | 193.051 | 1.92 | 178, 149, 134 | - | - | - | - | - | Organic acids | PCC, T |
| P49 | Dasycarpamin | $C_{17}H_{21}NO_4$ | 12.16 | - | - | - | - | - | [M+H]+ | 304.1543 | 304.1546 | 0.89 | 286, 271,256,232 | Alkaloids | PCC |
| P50 | Noroxyhydrastinine | $C_{10}H_9NO_3$ | 12.24 | - | - | - | - | - | [M+H]+ | 192.0655 | 192.0656 | 0.42 | 192, 163, 149, 119, 91 | Alkaloids | PCC |
| P51 | Demethyleneberberine | $C_{19}H_{18}NO_4^+$ | 12.29 | - | - | - | - | - | [M]+ | 324.1230 | 324.1233 | 0.83 | 309, 308, 280,266 | Alkaloids | PCC |
| P52 | Tribulusaponin A | $C_{45}H_{74}O_{20}$ | 12.3 | [M-H]- | 933.47007 | 933.4695 | -0.61 | 771 | - | - | - | - | - | Steroid saponins | T |

| | | | | | | | | | | | | | | | |
|---|---|---|---|---|---|---|---|---|---|---|---|---|---|---|---|
| P53 | Nicotiflorin | $C_{27}H_{30}O_{15}$ | 12.31 | [M-H]- | 593.1512 | 593.1494 | -3.02 | 549, 343, 325, 265, 207, 205, 181, 163, 151, 137 | - | - | - | - | - | Flavonoids | GF, T |
| P54 | 11-(6-O-trans-Sinapoylglucopyranosyl)gardendiol | $C_{27}H_{34}O_{13}$ | 12.34 | [M-H]- | 565.1927 | 565.1912 | -2.58 | 385, 357, 325, 295, 265, 223, 205 | - | - | - | - | - | Steroid saponins | GF |
| P55 | Hecogenin 3-O-β-glucopyranosyl(1→2)-β-glucopyranosyl(1→4)-galactopyranoside | $C_{45}H_{72}O_{19}$ | 12.34 | - | - | - | - | - | [M+H]+ | 917.4741 | 917.4733 | -0.83 | 755, 737, 593, 431 | Steroid saponins | T |
| P56 | 3,4-Dicaffeoyl quinic acid/3,5-Dicaffeoyl quinic acid/4,5-Dicaffeoyl quinic acid | $C_{25}H_{24}O_{12}$ | 12.51 | [M-H]- | 515.1195 | 515.1176 | -3.69 | 353, 191, 179, 135 | - | - | - | - | - | Organic acids | GF |
| P57 | Jasminoside R | $C_{22}H_{34}O_{12}$ | 12.51 | - | - | - | - | - | [M+Na]+ | 513.1943 | 513.1929 | -2.63 | 347 | Glycosides | GF |
| P58 | Oxyberberine | $C_{20}H_{17}NO_5$ | 12.58 | - | - | - | - | - | [M+H]+ | 352.1180 | 352.1182 | 0.71 | 337, 336, 322, 308, 294 | Alkaloids | PCC |

| | | | | | | | | | | | | | | | |
|---|---|---|---|---|---|---|---|---|---|---|---|---|---|---|---|
| P59 | Terrestrosin G | $C_{51}H_{86}O_{25}$ | 12.6 | [M-H]- | 1097.5385 | 1097.5388 | 0.24 | 935 | - | - | - | - | - | Steroid saponins | T |
| P60 | Crocin I or its isomer | $C_{44}H_{64}O_{24}$ | 12.61/14.79 | [M-H]- | 975.3715 | 975.3684 | -3.16 | 651, 327, 283, 179 | - | - | - | - | - | Glycosides | GF |
| P61 | 6"-O-p-Coumaroyl genipin gentiobioside | $C_{32}H_{40}O_{17}$ | 12.67 | [M-H]- | 695.2193 | 695.2191 | -0.24 | 663, 619, 469, 409, 367, 325, 307, 365, 225, 207, 163, 145, 123, 101 | - | - | - | - | - | Iridoids | GF |
| P62 | 6"-O-[trans-Sinapoyl] genipin gentiobioside | $C_{34}H_{44}O_{19}$ | 12.8 | [M-H]- | 755.2404 | 755.2404 | 0.00 | 723, 529, 427, 385, 225, 223, 205, 123, 101 | - | - | - | - | - | Iridoids | GF |
| P63 | Jasminoside Q/S | $C_{22}H_{36}O_{12}$ | 12.85 | [M+HCOO]- | 537.2189 | 537.2211 | 4.13 | 491, 323, 221, 179, 167, 119 | - | - | - | - | - | Glycosides | GF |
| P64 | 3,5-Di-O-caffeoyl-4-O-(3-hydroxy-3-methyl)-glutaroylquinic acid | $C_{31}H_{32}O_{16}$ | 13.03 | [M-H]- | 659.1618 | 659.1614 | -0.61 | 497, 435, 395, 353, 335, 273, 233, 191, 161 | - | - | - | - | - | Organic acids | GF |

| | | | | | | | | | | | | | | | |
|---|---|---|---|---|---|---|---|---|---|---|---|---|---|---|---|
| P65 | 3-Isobutylglutaric acid | $C_9H_{16}O_4$ | 13.17 | [M-H]- | 187.0976 | 187.0978 | 1.18 | 169, 144, 143, 125, 97 | - | - | - | - | - | Organic acids | PCC |
| P66 | Berberrubine | $C_{19}H_{16}ClNO_4$ | 13.32 | - | - | - | - | - | [M-Cl]+ | 322.1074 | 322.1080 | 1.86 | 307, 279 | Alkaloids | PCC |
| P67 | Columbamine/Jatrorrhizine | $C_{20}H_{20}NO_4+$ | 13.34 | - | - | - | - | - | [M]+ | 338.1387 | 338.1387 | 0.06 | 323,322, 308,307,306,294,280,277,265 | Alkaloids | PCC |
| P68 | 6'-O-trans-Sinapoyl jasminoside L | $C_{27}H_{36}O_{12}$ | 13.36 | [M-H]- | 551.2134 | 551.2112 | -3.99 | 533, 521, 385, 367, 325, 295, 265, 223, 205, 165 | - | - | - | - | - | Glycosides | GF |
| P69 | Crocin II | $C_{38}H_{54}O_{19}$ | 13.38 | [M+HCOO]- | 859.32413 | 859.3236 | -0.62 | 859, 813, 651, 327, 179 | [M+Na]+ | 837.3152 | 837.3144 | -0.90 | 675, 513,351 | Glycosides | GF |
| P70 | 4-Sinapoyl-5-caffeoylquinic acid | $C_{27}H_{28}O_{13}$ | 13.56 | [M-H]- | 559.1457 | 559.1454 | -0.55 | 397, 223, 173, 155 | - | - | - | - | - | Organic acids | GF |
| P71 | 6'-O-trans-Sinapoyl geniposide | $C_{28}H_{34}O_{14}$ | 13.65 | [M-H]- | 593.1876 | 593.1844 | -5.36 | 557, 225, 223, 205, 123, 101 | - | - | - | - | - | Iridoids | GF |
| P72 | Terrestrosin K | $C_{51}H_{82}O_{24}$ | 13.74 | [M-H]- | 1077.51233 | 1077.5118 | -0.49 | - | - | - | - | - | - | Steroid saponins | T |

| | | | | | | | | | | | | | | | |
|---|---|---|---|---|---|---|---|---|---|---|---|---|---|---|---|
| P73 | p-Coumaroyltyramine | $C_{17}H_{17}NO_3$ | 13.92 | - | - | - | - | - | [M+H]+ | 284.1281 | 284.1282 | 0.28 | 147, 121, 119, 103, 93, 91, 77 | Alkaloids | T |
| P74 | Terreside A | $C_{45}H_{72}O_{19}$ | 14.01 | [M-H]- | 915.4595 | 915.4578 | -1.86 | 753 | - | - | - | - | - | Steroid saponins | T |
| P75 | Palmatine | $C_{21}H_{22}NO_4+$ | 14.11 | - | - | - | - | - | [M]+ | 352.1543 | 352.1543 | -0.09 | 337,336,322,320, 308,294,292,278 | Alkaloids | PCC |
| P76 | N-trans-feruloyltyramine | $C_{18}H_{19}NO_4$ | 14.18 | [M+HCOO]- | 358.1296 | 358.1296 | -0.03 | 297, 190, 178, 148, 135 | - | - | - | - | - | Alkaloids | T |
| P77 | Berberine | $C_{20}H_{18}NO_4+$ | 14.21 | - | - | - | - | - | [M]+ | 336.1230 | 336.1237 | 1.99 | 321, 320, 318, 306, 304, 292, 291, 278, 275 | Alkaloids | PCC |
| P78 | 6''-O-trans-p-Cinnamoyl genipingentiobioside | $C_{33}H_{42}O_{18}$ | 14.29 | [M-H]- | 725.2298 | 725.2292 | -0.88 | 531, 355, 225, 207, 147, 123 | - | - | - | - | - | Iridoids | GF |
| P79 | Quercetin | $C_{15}H_{10}O_7$ | 14.62 | [M-H]- | 301.0354 | 301.0357 | 1.06 | 273, 229, 179, 151, 121 | - | - | - | - | - | Flavonoids | GF |
| P80 | 5,7,3',4'-Tetrahydroxy-6,8-dimethoxy flavone | $C_{17}H_{14}O_8$ | 14.82 | [M-H]- | 345.0616 | 345.0607 | -2.58 | 300, 315, 287, 284, 269 | - | - | - | - | - | Flavonoids | GF |

| | | | | | | | | | | | | | | | |
|---|---|---|---|---|---|---|---|---|---|---|---|---|---|---|---|
| P81 | 5-Hydroxy-1,7-bis(4-hydroxyphenyl)-3-heptanone | $C_{19}H_{22}O_4$ | 14.86 | [M-H]- | 313.14453 | 313.1446 | 0.22 | 298, 255, 163, 149 | - | - | - | - | - | Phenyls | CLR |
| P82 | Crocin III or its isomer | $C_{32}H_{44}O_{14}$ | 15.08/16.65 | [M-H]- | 651.2658 | 651.2653 | -0.81 | 327, 283, 239, 179, 143, 89 | - | - | - | - | - | Glycosides | GF |
| P83 | 2-(4,5-Dihydroxy-3-methoxyphenyl)methenyl-5-(4-hydroxyphenyl)ethenyl-3(2H)-furanone | $C_{20}H_{16}O_6$ | 15.16 | - | - | - | - | - | [M+H]+ | 353.1020 | 353.1015 | -1.30 | 290, 179, 147, 107 | Furanone | CLR |
| P84 | 2-(4,5-Dihydroxy-3-methoxyphenyl)methenyl-5-(4-hydroxy-3-methoxyphenyl)ethenyl-3(2H)-furanone | $C_{21}H_{18}O_7$ | 15.35 | - | - | - | - | - | [M+H]+ | 383.1125 | 383.1126 | 0.18 | 368, 294, 177 | Furanone | CLR |
| P85 | Rutaevin | $C_{26}H_{30}O_9$ | 15.81 | [M+HCOO]- | 531.18719 | 531.1871 | -0.17 | 469, 467, 423, 411, 261, 233, 177, 175 | - | - | - | - | - | Limonoids | PCC |
| P86 | Skimmianine | $C_{14}H_{13}NO_4$ | 15.99 | - | - | - | - | - | [M+H]+ | 260.0917 | 260.0933 | 6.04 | 260, 227, 199, 136 | Alkaloids | PCC |
| P87 | Gamma-Fagarine | $C_{13}H_{11}NO_3$ | 16.15 | - | - | - | - | - | [M+H]+ | 230.0812 | 230.0817 | 2.30 | 215, 214, 200,186,172 | Alkaloids | PCC |

| | | | | | | | | | | | | | | |
|---|---|---|---|---|---|---|---|---|---|---|---|---|---|---|
| P88 | Tribulusamide A | $C_{36}H_{36}N_2O_8$ | 16.18 | [M-H]- | 623.2399 | 623.2397 | -0.30 | 460, 445, 432, 297 | [M+H]+ | 625.2544 | 625.255 | 0.90 | 488, 462, 351, 325, 308, 307, 297, 265, 201, 164, 121 | Alkaloids | T |
| P89 | 1,5-Bis(4-hydroxy-3-methoxyphenyl)penta-1,4-dien-3-one | $C_{19}H_{18}O_5$ | 16.29 | - | - | - | - | - | [M+H]+ | 327.1227 | 327.1235 | 2.45 | 277, 203, 177, 145, 137 | Ketone | CLR |
| P90 | Chloromaloside A | $C_{50}H_{80}O_{23}$ | 17.07 | [M+HCOO]- | 1093.50724 | 1093.5052 | -1.87 | 1047, 915, 885, 591, 322 | - | - | - | - | - | Steroid saponins | T |
| P91 | 1-(3,4-Dihydroxyphenyl)-7-(4-hydroxy-3-methoxyphenyl)hepta-1,6-diene-3,5-dione | $C_{20}H_{18}O_6$ | 17.25 | - | - | - | - | - | [M+H]+ | 355.1176 | 355.1180 | 1.10 | 271, 239, 211,177,163,147, 145, 137,135 | Ketone | CLR |
| P92 | Limonin | $C_{26}H_{30}O_8$ | 17.26 | [M-H]- | 469.18679 | 469.1867 | -0.19 | 487, 471, 435, 425, 411, 407, 381, 365, 349, 331, 233, 229 | [M+H]+ | 471.2013 | 471.2013 | -0.08 | 435, 425, 407,367,161 | Limonoids | PCC |
| P93 | Tetrahydrobisdemethoxycurcumin | $C_{19}H_{20}O_4$ | 17.73 | [M-H]- | 311.12888 | 311.129 | 0.39 | 205,163,99, 57 | [M+H]+ | 313.1434 | 313.1428 | -2.04 | 107 | Curcumin | CLR |

| | | | | | | | | | | | | | | | |
|---|---|---|---|---|---|---|---|---|---|---|---|---|---|---|---|
| P94 | Dihydrobisdemethoxycurcumin | $C_{19}H_{18}O_4$ | 17.91 | [M-H]- | 309.11323 | 309.1133 | 0.23 | 119, 189, | [M+H]+ | 311.1278 | 311.1285 | 2.28 | 147, 205, 107 | Curcumin | CLR |
| P95 | Bisdemethoxycurcumin | $C_{19}H_{16}O_4$ | 18.02 | [M-H]- | 307.09758 | 307.0976 | 0.07 | 143, 119 | [M+H]+ | 309.1121 | 309.1132 | 3.56 | 225, 147, 131, 119, 91 | Curcumin | CLR |
| P96 | Dihydrodemethoxycurcumin | $C_{20}H_{20}O_5$ | 18.14 | [M-H]- | 339.1238 | 339.1238 | 0.00 | 119, 189, 149, 219, 337, 339 | [M+H]+ | 341.1384 | 341.1388 | 1.17 | 147, 137, 122, 119 | Curcumin | CLR |
| P97 | Demethoxycurcumin | $C_{20}H_{18}O_5$ | 18.268 | [M-H]- | 337.10815 | 337.1081 | -0.15 | 217, 202, 187,173,158,149, 143,134, 119 | [M+H]+ | 339.1227 | 339.1228 | 0.29 | 255,233,195,177,161,147,145,131,119,91 | Curcumin | CLR |
| P98 | Dioscin | $C_{45}H_{72}O_{16}$ | 18.29 | - | - | - | - | - | [M+H]+ | 869.4893 | 869.4897 | 0.45 | 689, 671, 653, 455, 437, 419, 217, 157, 143, 125, 97 | Steroid saponins | T |
| P99 | Dihydrocurcumin | $C_{21}H_{22}O_6$ | 18.38 | [M-H]- | 369.13436 | 369.1349 | 1.46 | 219, 149, 134 | [M+H]+ | 371.1489 | 371.1492 | 0.78 | 177, 145, 137, 117 | Curcumin | CLR |
| P100 | Curcumin | $C_{21}H_{20}O_6$ | 18.51 | [M-H]- | 367.11871 | 367.1187 | -0.03 | 217, 173, 149, 134 | [M+H]+ | 369.1333 | 369.1347 | 3.90 | 285,253,225,177, 145,117 | Curcumin | CLR |
| P101 | Tribulosin | $C_{55}H_{90}O_{25}$ | 19.27 | [M+HCOO]- | 1195.5753 | 1195.5774 | 1.74 | 1017 | - | - | - | - | - | Steroid saponins | T |
| P102 | 7-(3,4-Dimethoxyphenyl)-1-(4-hydroxy-3- | $C_{22}H_{24}O_6$ | 19.42 | - | - | - | - | - | [M+H]+ | 385.1646 | 385.1647 | 0.36 | 193, 177,151, 145,117 | Ketone | CLR |

| | | | | | | | | | | | | | | | |
|---|---|---|---|---|---|---|---|---|---|---|---|---|---|---|---|
| | methoxyphenyl)hept-1-ene-3,5-dione | | | | | | | | | | | | | | |
| P103 | 1-(3,4-Dimethoxyphenyl)-7-(4-hydroxy-3-methoxyphenyl)hepta-1,6-diene-3,5-dione | $C_{22}H_{22}O_6$ | 19.74 | - | - | - | - | - | [M+H]+ | 383.1489 | 383.1496 | 1.80 | 299,273,268,259,239,219,191,177, 175,160,145, 132,117 | Ketone | CLR |
| P104 | Caryophyllene Oxide | $C_{15}H_{24}O$ | 22.21 | - | - | - | - | - | [M+H]+ | 221.1900 | 221.1900 | 0.05 | 159,149,137,123,119,111,95,91,81,69,67,55 | Alkanes | PCC |
| P105 | Ursolic acid | $C_{30}H_{48}O_3$ | 23.86 | [M-H]- | 455.3531 | 455.3521 | -2.13 | 455 | [M+H]+ | 457.3676 | 457.3681 | 1.05 | 411 | Triterpenoids | GF |
| P106 | Glyceryl palmitate | $C_{19}H_{38}O_4$ | 23.93 | - | - | - | - | - | [M+H]+ | 331.2843 | 331.2846 | 0.94 | 313, 257, 239, 109, 99, 95, 81, 71, 57 | Organic acids | T |

Table S2 prototypic and metabolic components of representative compounds of XRG-4

| Class | Prototype NO. | Prototype | Metabolite | Biotransformation | Formula | m/z | ppm | R.T. (min) | % Score |
|---|---|---|---|---|---|---|---|---|---|
| **Alkaloids** | P77 | Berberine | P51 | Demethyleneberberine (Loss of $CH_2$ and Hydrogenation) | $C_{19}H_{18}NO_4$ | 324.1230 | -0.1 | 12.30 | 72.1 |
| | | | M1 | Demethyleneberberine and Glucuronidation | $C_{25}H_{26}NO_{10}$ | 500.1551 | 0.0 | 11.08 | 73.7 |
| | | | M2 | Demethyleneberberine and Di-Glucuronidation | $C_{31}H_{34}NO_{16}$ | 676.1873 | 0.1 | 9.83 | 75.2 |
| | | | M3 | Hydrogenation and Glucuronidation | $C_{26}H_{28}NO_{10}$ | 514.1707 | -0.1 | 11.16 | 76.2 |
| | | | M4 | Loss of $CH_2$ | $C_{19}H_{16}NO_4$ | 322.1073 | -0.2 | 13.31 | 72.5 |
| | | | M5 | Loss of $CH_2$ and Glucuronidation | $C_{25}H_{24}NO_{10}$ | 498.1394 | -0.1 | 11.53 | 72.5 |
| | | | M6 | Loss of $CH_2$ and Sulfate Conjugation | $C_{19}H_{16}NO_7S$ | 402.0632 | -2.5 | 13.51 | 72.2 |
| **Curcumin** | P100 | Curcumin | M7 | Loss of $CH_2$ and $CH_2O$ | $C_{19}H_{16}O_5$ | 323.0908 | -5.2 | 10.99 | 66.9 |
| **Flavonoids** | P46 | Isoquercetin/Hyperoside | M8 | Loss of $C_{15}H_8O_7$+Demethylation to Carboxylic Acid | $C_6H_{10}O_7$ | 193.0351 | -1.3 | 14.77 | 75.0 |
| | | | M9 | Loss of $C_6H_{10}O_6$+Hydrogenation | $C_{15}H_{12}O_6$ | 287.0584 | 7.9 | 13.33 | 60.3 |
| | | | M10 | Loss of $C_6H_{10}O_6$ | $C_{15}H_{10}O_6$ | 285.0395 | -3.4 | 14.54 | 71.6 |
| | P42 | Rutin | M8 | Loss of $C_{21}H_{18}O_{11}$+Demethylation to Carboxylic Acid | $C_6H_{10}O_7$ | 193.0351 | -1.3 | 14.77 | 75.0 |
| | | | M10 | Loss of $C_{12}H_{20}O_{10}$ | $C_{15}H_{10}O_6$ | 285.0395 | -3.4 | 14.54 | 71.6 |
| | | | M11 | Loss of O and $C_6H_{10}O_6$+Hydrogenation | $C_{21}H_{22}O_9$ | 417.1177 | -3.5 | 13.54 | 71.3 |
| **Iridoids** | P30 | Geniposide | M12 | Loss of $C_7H_{12}O_8$+Demethylation | $C_{10}H_{12}O_4$ | 195.0661 | -1.1 | 12.29 | 76.7 |

| | | | | | | | | |
|---|---|---|---|---|---|---|---|---|
| | | M13 | Loss of $C_7H_{12}O_7$+Methylation | $C_{12}H_{16}O_5$ | 239.0923 | -0.7 | 12.59 | 75.0 |
| | | M14 | Loss of $CH_2O_3$ and $C_2H_4O_3$ | $C_{15}H_{20}O_6$ | 295.1177 | -3.6 | 14.19 | 77.9 |
| | | M15 | Loss of $CH_2O_3$ and $C_2H_4O_3$ | $C_{15}H_{20}O_6$ | 295.1178 | -3.2 | 13.17 | 75.2 |
| | | M16 | Loss of $C_7H_{12}O_7$+Sulfate Conjugation | $C_{11}H_{14}O_8S$ | 305.0334 | -0.8 | 10.11 | 61.8 |
| | | M17 | Loss of $CH_2O_3$ and O+Loss of Hydroxymethylene | $C_{16}H_{22}O_7$ | 325.1285 | -2.3 | 13.60 | 75.1 |
| | | M18 | Loss of $CH_2O_3$ and O+Loss of Hydroxymethylene | $C_{16}H_{22}O_7$ | 325.1285 | -2.5 | 14.06 | 76.8 |
| | | M19 | Loss of $CH_2O_3$+Loss of Hydroxymethylene | $C_{16}H_{22}O_8$ | 341.1225 | -4.8 | 14.18 | 74.6 |
| | | M20 | Loss of $CH_2O_3$+Loss of Hydroxymethylene | $C_{16}H_{22}O_8$ | 341.1228 | -4 | 14.38 | 73.1 |
| | | M21 | Loss of $C_2H_4O_3$ | $C_{16}H_{22}O_9$ | 357.1173 | -5.1 | 10.37 | 69.4 |
| | | M22 | Loss of $C_2H_4O_3$ | $C_{16}H_{22}O_9$ | 357.1183 | -2.3 | 11.41 | 77.9 |
| | | M23 | Loss of $C_2H_4O_3$+Hydrogenation | $C_{16}H_{24}O_9$ | 359.1336 | -3.3 | 10.79 | 72.1 |
| | | M24 | Loss of $C_2H_4O_3$+Hydrogenation | $C_{16}H_{24}O_9$ | 359.1338 | -2.6 | 8.98 | 74.0 |
| | | M25 | Loss of $C_2H_4O_2$ | $C_{16}H_{22}O_{10}$ | 373.112 | -5.4 | 10.64 | 69.4 |
| | | M26 | Loss of $CH_2O_3$+Demethylation to Carboxylic Acid | $C_{17}H_{22}O_{11}$ | 401.1077 | -3.1 | 12.79 | 77.7 |
| P20 | Genipin 1-gentiobioside | P30 | Glucoside hydrolysis | $C_{17}H_{24}O_{10}$ | 387.1296 | -0.2 | 10.17 | 81.2 |
| | | M26 | Glucoside hydrolysis+Demethylation to Carboxylic Acid | $C_{17}H_{22}O_{11}$ | 401.1077 | -3.1 | 12.79 | 77.7 |
| | | M25 | Glucoside hydrolysis and Loss of $CH_2$ | $C_{16}H_{22}O_{10}$ | 373.112 | -5.4 | 10.64 | 69.4 |

| | | | | | | | | |
|---|---|---|---|---|---|---|---|---|
| | | | M16 | Glucoside hydrolysis +Sulfate Conjugation | $C_{11}H_{14}O_8S$ | 305.0334 | -0.8 | 10.11 | 61.8 |
| **Glycosides (Monoterpenoids)** | P14 | Jasminoside B/F | M8 | Loss of $C_{10}H_{14}O_3$+Demethylation to Carboxylic Acid | $C_6H_{10}O_7$ | 193.0351 | -1.3 | 14.77 | 75.0 |
| | | | M23 | Ketone Formation | $C_{16}H_{24}O_9$ | 359.1336 | -3.3 | 10.79 | 72.6 |
| | | | M24 | Ketone Formation | $C_{16}H_{24}O_9$ | 359.1338 | -2.6 | 8.98 | 75.4 |
| | | | M27 | Loss of $C_6H_{10}O_6$+Methylation | $C_{11}H_{18}O_2$ | 181.1234 | 0 | 14.65 | 75.0 |
| | | | M28 | Loss of $C_6H_{10}O_5$ | $C_{10}H_{16}O_3$ | 183.1027 | 0.3 | 9.48 | 80.2 |
| | | | M29 | Loss of $C_6H_{10}O_5$+Oxidation | $C_{10}H_{16}O_4$ | 199.0977 | 0.8 | 8.72 | 76.3 |
| | | | M30 | Loss of $C_6H_{10}O_5$+Demethylation to Carboxylic Acid | $C_{10}H_{14}O_5$ | 213.0769 | 0.3 | 7.71 | 76.2 |
| | | | M31 | Loss of O and O+Loss of Hydroxymethylene | $C_{15}H_{24}O_5$ | 283.1538 | -4.6 | 14.99 | 49.1 |
| | | | M32 | Loss of O and O+Loss of Hydroxymethylene | $C_{15}H_{24}O_5$ | 283.1543 | -3 | 15.17 | 53.0 |
| | | | M33 | Loss of O and O+Loss of Hydroxymethylene | $C_{15}H_{24}O_5$ | 283.1543 | -3 | 15.46 | 48.2 |
| | | | M34 | Loss of O+Loss of Hydroxymethylene | $C_{15}H_{24}O_6$ | 299.149 | -3.5 | 12.71 | 74.2 |
| | | | M35 | Loss of O and O+Hydrogenation | $C_{16}H_{28}O_6$ | 315.18 | -4.1 | 12.17 | 73.6 |
| | | | M36 | Loss of O and O+Hydrogenation | $C_{16}H_{28}O_6$ | 315.1806 | -2.2 | 11.55 | 75.2 |
| | | | M37 | Loss of O | $C_{16}H_{26}O_7$ | 329.1604 | -0.5 | 9.07 | 78.6 |
| | | | M38 | Loss of O+Hydrogenation | $C_{16}H_{28}O_7$ | 331.1748 | -4.5 | 10.42 | 68.9 |
| | | | M39 | Loss of O+Hydrogenation | $C_{16}H_{28}O_7$ | 331.1749 | -4 | 10.82 | 70.9 |
| | | | M40 | Loss of O+Hydrogenation | $C_{16}H_{28}O_7$ | 331.1755 | -2.1 | 10.27 | 75.3 |
| | | | M41 | Oxidation | $C_{16}H_{26}O_9$ | 361.1493 | -3.1 | 11.14 | 73.4 |

| | | | | | | | | | |
|---|---|---|---|---|---|---|---|---|---|
| | | | M42 | Phosphorylation | $C_{16}H_{27}O_{11}P$ | 425.1249 | 7.3 | 12.73 | 63.4 |
| | | | M43 | Desaturation | $C_{16}H_{24}O_8$ | 343.1385 | -3.8 | 11.01 | 74.6 |
| | | | M44 | Desaturation | $C_{16}H_{24}O_8$ | 343.1388 | -2.9 | 14.57 | 75.6 |
| | | | P44 | Loss of $C_6H_{10}O_5$ | $C_{10}H_{16}O_3$ | 183.1027 | 0.3 | 11.58 | 80.2 |
| **Organic acids** | P65 | 3-Isobutylglutaric acid | M45 | Desaturation | $C_9H_{14}O_4$ | 185.0822 | 1.6 | 8.03 | 75.6 |
| | P36 | 3-O-Feruloylquinic acid | M46 | Loss of $C_7H_{10}O_5$ | $C_{10}H_{10}O_4$ | 193.0512 | 3 | 9.92 | 73.2 |
| | | | M47 | Loss of $CH_2$ and $C_7H_{10}O_6$+Sulfate Conjugation | $C_9H_8O_6S$ | 242.9986 | 6.9 | 9.82 | 38.9 |
| | | | M48 | Loss of $CH_2$ and $C_7H_{10}O_5$+Sulfate Conjugation | $C_9H_8O_7S$ | 258.9919 | 0.2 | 9.76 | 58.7 |
| | | | M49 | Loss of $CH_2$ and $C_7H_{10}O_5$+Oxidation | $C_9H_8O_5$ | 195.0306 | 3.4 | 12.30 | 71.5 |
| | P70 | 4-Sinapoyl-5-caffeoylquinic acid | M46 | Loss of $CH_2$ and $C_{16}H_{16}O_9$ | $C_{10}H_{10}O_4$ | 193.0512 | 3 | 9.92 | 72.5 |
| | | | M47 | Loss of $C_{18}H_{20}O_{10}$+Sulfate Conjugation | $C_9H_8O_6S$ | 242.9986 | 6.9 | 9.82 | 37.7 |
| | | | M48 | Loss of $C_{18}H_{20}O_9$+Sulfate Conjugation | $C_9H_8O_7S$ | 258.9919 | 0.2 | 9.76 | 50.0 |
| | | | M50 | Loss of $C_{18}H_{20}O_{10}$+Glycine Conjugation | $C_{11}H_{11}NO_4$ | 220.0614 | -0.8 | 10.84 | 75.0 |
| | | | M51 | Loss of $C_{16}H_{16}O_9$+Methylation | $C_{12}H_{14}O_4$ | 221.0816 | -1.6 | 14.23 | 75.0 |
| | | | M52 | Loss of $C_{16}H_{16}O_8$+Sulfate Conjugation | $C_{11}H_{12}O_8S$ | 303.0183 | 0.8 | 9.56 | 50.0 |
| | | | M53 | Loss of $C_{11}H_{10}O_5$+Hydrogenation | $C_{16}H_{20}O_8$ | 339.1069 | -4.7 | 13.93 | 68.2 |
| | | | M54 | Loss of $C_{11}H_{10}O_5$+Hydrogenation | $C_{16}H_{20}O_8$ | 339.1069 | -4.7 | 14.09 | 68.2 |

| | | | | | | | | | |
|---|---|---|---|---|---|---|---|---|---|
| | | | M55 | Loss of $CH_2$ and $CH_2$+Sulfate Conjugation | $C_{25}H_{24}O_{16}S$ | 611.0708 | -0.6 | 10.11 | 50.0 |
| | | | P36 | Loss of $CH_2$ and $C_9H_6O_4$ | $C_{17}H_{20}O_9$ | 367.1024 | -2.9 | 10.89 | 72.9 |
| **Steroid saponins** | P98 | Dioscin | M56 | Loss of $C_{27}H_{40}O_2$ and $C_6H_{10}O_5$ | $C_{12}H_{22}O_9$ | 311.1317 | -6.3 | 14.17 | 64.3 |

Table S 3　Distribution of substance basis in vivo

| **NO.** | **Name** | **Ptoyotypes** | **Urine** | **Plasma** | **Feces** | **Brain** | **Heart** | **Kidney** | **Liver** | **Lung** | **Spleen** |
|---|---|---|---|---|---|---|---|---|---|---|---|
| 1 | Quinic acid | P1 | √ | √ | √ | - | - | - | - | - | - |
| 2 | Candicine | P3 | - | - | √ | - | - | - | - | - | - |
| 3 | Gardoside | P4 | √ | - | - | - | - | - | - | - | - |
| 4 | Geniposidic acid | P6 | √ | - | - | - | - | - | - | - | - |
| 5 | Shazhiside or its isomer | P8 | √ | - | - | - | - | - | - | - | - |
| 6 | Gentisic acid | P10 | - | - | √ | - | - | - | - | - | - |
| 7 | Feretoside | P12 | √ | - | - | - | - | - | - | - | - |
| 8 | Jasminoside D/G | P16 | √ | - | - | - | - | - | - | - | - |
| 9 | Genipin 1-gentiobioside | P20 | √ | √ | - | - | - | - | - | - | - |
| 10 | Clausenamide | P21 | √ | - | √ | - | - | - | - | - | - |
| 11 | 5-O-Feruloylquinic acid | P23 | √ | - | - | - | - | - | - | - | - |
| 12 | Phellodendrine oxide | P26 | √ | - | √ | - | - | - | - | - | - |
| 13 | N-Methylhigenamine 7-glucopyranoside | P27 | √ | - | - | - | - | - | - | - | - |
| 14 | Tetrahydrojatrorrhizine | P28 | √ | - | √ | - | - | - | - | - | - |
| 15 | Caffeic acid | P29 | √ | - | √ | - | - | - | - | - | - |
| 16 | Geniposide | P30 | √ | √ | - | - | - | - | - | - | - |
| 17 | Tembetarine | P31 | - | - | √ | - | - | - | - | - | - |

| | | | | | | | | | | | |
|---|---|---|---|---|---|---|---|---|---|---|---|
| 18 | Phellodendrine | P33 | √ | - | √ | - | - | - | - | - | - |
| 19 | Picrocrocin | P34 | √ | - | - | - | - | - | - | - | - |
| 20 | 3-O-Feruloylquinic acid | P36 | √ | - | - | - | - | - | - | - | - |
| 21 | Lotusine | P38 | - | - | √ | - | - | - | - | - | - |
| 22 | 4-O-Feruloylquinic acid | P41 | √ | - | - | - | - | - | - | - | - |
| 23 | Jasminodiol | P44 | √ | - | √ | - | - | - | - | - | - |
| 24 | Menisperine | P45 | - | - | √ | - | - | - | - | - | - |
| 25 | Ferulic acid | P48 | √ | √ | √ | - | - | - | - | - | - |
| 26 | Demethyleneberberine | P51 | √ | - | √ | - | - | - | - | - | - |
| 27 | Oxyberberine | P58 | - | - | √ | - | - | - | - | - | - |
| 28 | Berberrubine | P66 | √ | - | √ | - | - | - | √ | - | - |
| 29 | Columbamine/Jatrorrhizine | P67 | - | - | √ | - | √ | √ | - | - | - |
| 30 | Palmatine | P75 | - | - | √ | - | √ | √ | - | - | - |
| 31 | Berberine | P77 | - | √ | √ | √ | √ | √ | √ | √ | √ |
| 32 | Rutaevin | P85 | √ | - | - | - | - | - | - | - | - |
| 33 | Curcumin | P100 | √ | - | - | - | - | - | - | - | - |
| | **Count** | | **24** | **5** | **19** | **1** | **3** | **3** | **2** | **1** | **1** |
| 34 | M1 | | √ | - | - | - | - | - | √ | - | - |
| 35 | M2 | | √ | - | - | - | - | - | - | - | - |
| 36 | M3 | | √ | - | - | - | - | - | - | - | - |
| 37 | M4 | | √ | - | √ | - | - | - | - | - | - |
| 38 | M5 | | √ | - | - | - | - | - | - | - | - |
| 39 | M6 | | √ | - | - | - | - | - | - | - | - |
| 40 | M7 | | √ | - | - | - | - | - | - | - | - |
| 41 | M8 | | √ | - | - | - | - | - | - | - | - |

| | | | | | | | | | | |
|---|---|---|---|---|---|---|---|---|---|---|
| 42 | M9 | √ | - | - | - | - | - | - | - | - |
| 43 | M10 | | - | √ | - | - | - | - | - | - |
| 44 | M11 | √ | - | - | - | - | - | - | - | - |
| 45 | M12 | √ | - | - | - | - | - | - | - | - |
| 46 | M13 | √ | - | - | - | - | - | - | - | - |
| 47 | M14 | √ | - | - | - | - | - | - | - | - |
| 48 | M15 | √ | - | - | - | - | - | - | - | - |
| 49 | M16 | √ | √ | - | - | - | - | - | √ | - |
| 50 | M17 | √ | - | - | - | - | - | √ | - | - |
| 51 | M18 | √ | - | - | - | - | - | - | - | - |
| 52 | M19 | √ | - | - | - | - | - | - | - | - |
| 53 | M20 | √ | - | - | - | - | - | - | - | - |
| 54 | M21 | √ | - | - | - | - | - | - | - | - |
| 55 | M22 | √ | - | - | - | - | - | - | - | - |
| 56 | M23 | √ | - | - | - | - | - | - | - | - |
| 57 | M24 | √ | - | - | - | - | - | - | - | - |
| 58 | M25 | √ | - | - | - | - | - | - | - | - |
| 59 | M26 | √ | - | - | - | - | - | - | - | - |
| 60 | M27 | √ | - | - | - | - | - | - | - | - |
| 61 | M28 | √ | - | √ | - | - | - | - | - | - |
| 62 | M29 | √ | - | - | - | - | - | - | - | - |
| 63 | M30 | √ | - | - | - | - | - | - | - | - |
| 64 | M31 | √ | - | √ | - | - | - | - | - | - |
| 65 | M32 | √ | - | √ | - | - | - | - | - | - |
| 66 | M33 | √ | - | √ | - | - | - | - | - | - |

| | | | | | | | | | | |
|---|---|---|---|---|---|---|---|---|---|---|
| 67 | M34 | √ | - | - | - | - | - | - | - | - |
| 68 | M35 | √ | - | - | - | - | - | - | - | - |
| 69 | M36 | √ | - | - | - | - | - | - | - | - |
| 70 | M37 | √ | - | - | - | - | - | - | - | - |
| 71 | M38 | √ | - | - | - | - | - | - | - | - |
| 72 | M39 | √ | - | - | - | - | - | - | - | - |
| 73 | M40 | √ | - | - | - | - | - | - | - | - |
| 74 | M41 | √ | - | - | - | - | - | - | - | - |
| 75 | M42 | √ | - | - | - | - | - | - | - | - |
| 76 | M43 | √ | - | - | - | - | - | √ | - | - |
| 77 | M44 | √ | - | - | - | - | - | - | - | - |
| 78 | M45 | √ | - | - | - | - | - | - | - | - |
| 79 | M46 | √ | - | - | - | - | - | - | - | - |
| 80 | M47 | √ | √ | - | - | - | - | - | - | - |
| 81 | M48 | √ | √ | - | - | - | - | - | - | - |
| 82 | M49 | | - | √ | - | - | - | - | - | - |
| 83 | M50 | √ | - | - | - | - | - | - | - | - |
| 84 | M51 | √ | - | - | - | - | - | - | - | - |
| 85 | M52 | √ | - | - | - | - | - | - | - | - |
| 86 | M53 | √ | √ | - | - | - | - | - | - | - |
| 87 | M54 | √ | - | - | - | - | - | - | - | - |
| 88 | M55 | √ | - | - | - | - | - | - | - | - |
| 89 | M56 | √ | - | - | - | - | - | - | - | - |
| | **Count** | **54** | **4** | **7** | **0** | **0** | **0** | **3** | **1** | **0** |
| | **All** | **78** | **9** | **26** | **1** | **3** | **3** | **5** | **2** | **1** |

"√" indicates detection

Figure S1 The base peak chromatograms of XRG-4 in plasma

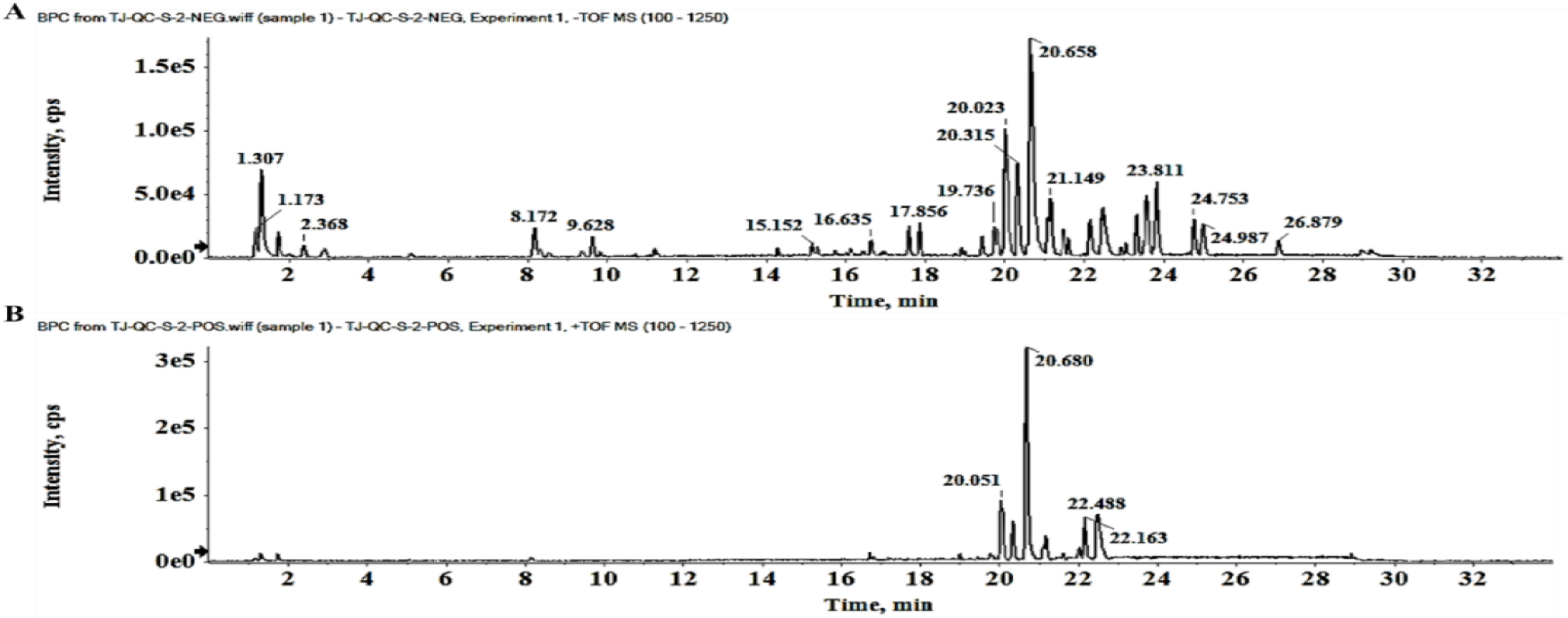


Figure S 1. the base peak chromatograms of XRG-4 in Plasma, (A) negative ion (B) positive ion

Figure S 2 The base peak chromatograms of XRG-4 in urine

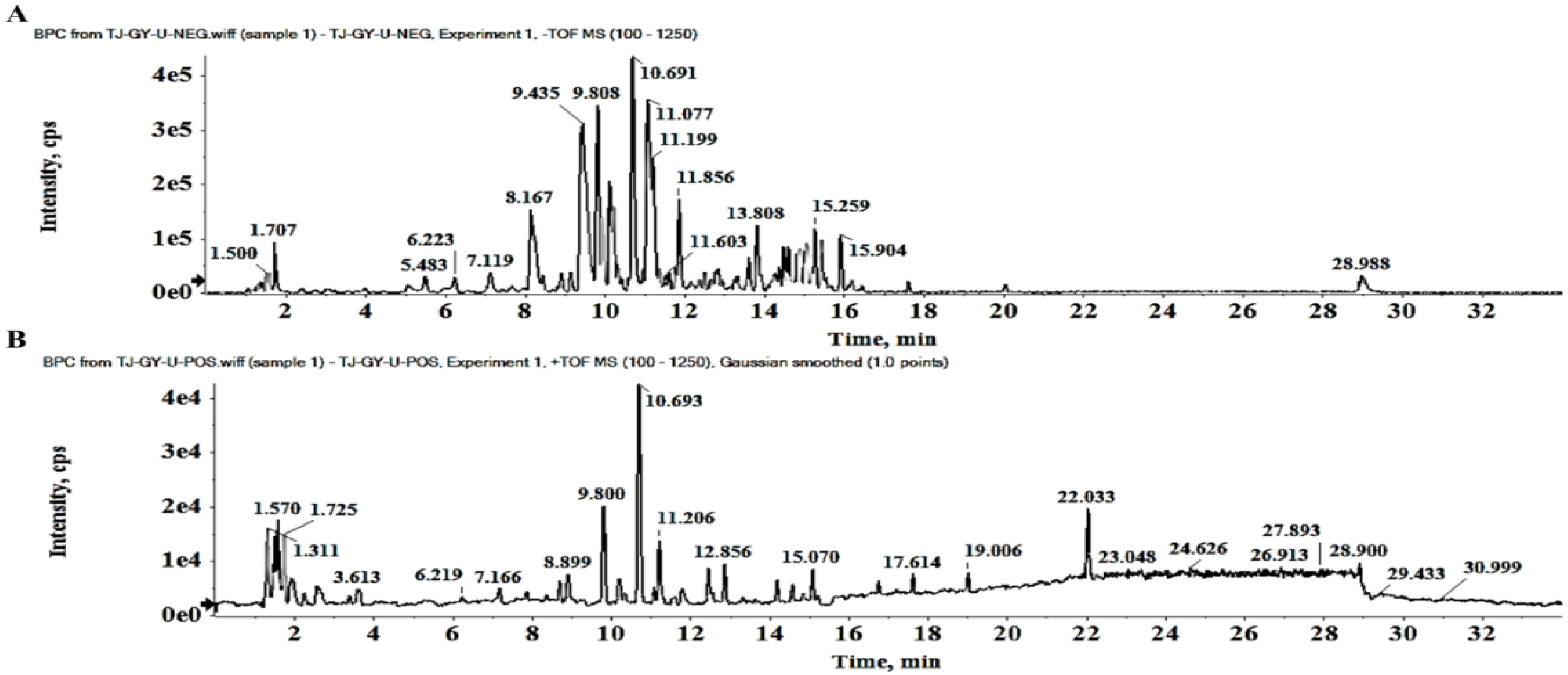


Figure S 2　the base peak chromatograms of XRG-4 in urine，(A) negative ion (B) positive ion

Figure S 3 The base peak chromatograms of XRG-4 in feces

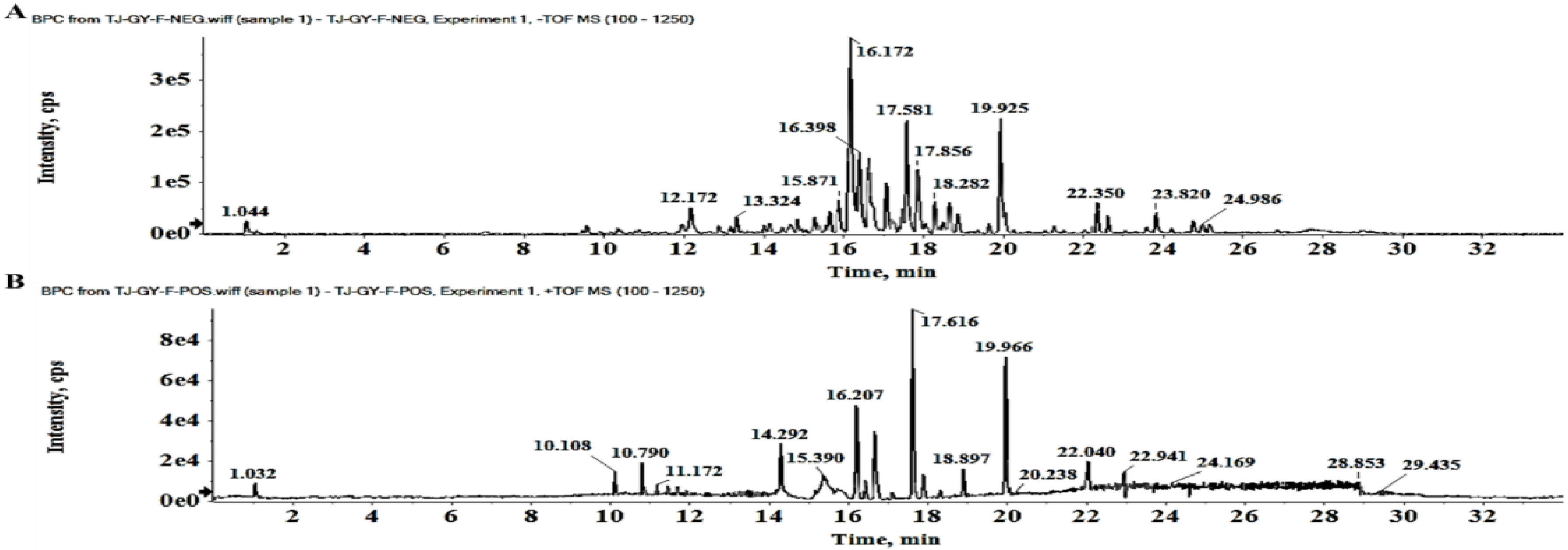


Figure S3, the base peak chromatograms of XRG-4 in feces, (A) negative ion (B) positive ion
