# Supplementary material for: Chemicolome and Metabolome Profiling of Xieriga-4 Decoction, A Traditional Mongolian Medicine, Using the UPLC-QTOF/MS Approach
Source: Evid Based Complement Alternat Med. 2022 Nov 16;2022:8197364. doi: 10.1155/2022/8197364 (PMC9683986; doi:10.1155/2022/8197364)
Supplement: Supplementary Materials — Supplementary information available: Tables S1-S3 and Figures S1-S3. [file 8197364.f1.zip › Table S2 (1).docx]

Table S2 prototypic and metabolic components of representative compounds of XRG-4

| **Class** | **Prototype NO.** | **Prototype** | **Metabolite** | **Biotransformation** | **Formula** | **m/z** | **ppm** | **R.T. (min)** | **% Score** |
| --- | --- | --- | --- | --- | --- | --- | --- | --- | --- |
| **Alkaloids** | P77 | Berberine | P51 | Demethyleneberberine (Loss of CH_2_ and Hydrogenation) | C_19_H_18_NO_4_ | 324.1230 | -0.1 | 12.30 | 72.1 |
|  |  |  | M1 | Demethyleneberberine and Glucuronidation | C_25_H_26_NO_10_ | 500.1551 | 0.0 | 11.08 | 73.7 |
|  |  |  | M2 | Demethyleneberberine and Di-Glucuronidation | C_31_H_34_NO_16_ | 676.1873 | 0.1 | 9.83 | 75.2 |
|  |  |  | M3 | Hydrogenation and Glucuronidation | C_26_H_28_NO_10_ | 514.1707 | -0.1 | 11.16 | 76.2 |
|  |  |  | M4 | Loss of CH_2_ | C_19_H_16_NO_4_ | 322.1073 | -0.2 | 13.31 | 72.5 |
|  |  |  | M5 | Loss of CH_2_ and Glucuronidation | C_25_H_24_NO_10_ | 498.1394 | -0.1 | 11.53 | 72.5 |
|  |  |  | M6 | Loss of CH_2_ and Sulfate Conjugation | C_19_H_16_NO_7_S | 402.0632 | -2.5 | 13.51 | 72.2 |
| **Curcumin** | P100 | Curcumin | M7 | Loss of CH_2_ and CH_2_O | C_19_H_16_O_5_ | 323.0908 | -5.2 | 10.99 | 66.9 |
| **Flavonoids** | P46 | Isoquercetin/Hyperoside | M8 | Loss of C_15_H_8_O_7_+Demethylation to Carboxylic Acid | C_6_H_10_O_7_ | 193.0351 | -1.3 | 14.77 | 75.0 |
|  |  |  | M9 | Loss of C_6_H_10_O_6_+Hydrogenation | C_15_H_12_O_6_ | 287.0584 | 7.9 | 13.33 | 60.3 |
|  |  |  | M10 | Loss of C_6_H_10_O_6_ | C_15_H_10_O_6_ | 285.0395 | -3.4 | 14.54 | 71.6 |
|  | P42 | Rutin | M8 | Loss of C_21_H_18_O_11_+Demethylation to Carboxylic Acid | C_6_H_10_O_7_ | 193.0351 | -1.3 | 14.77 | 75.0 |
|  |  |  | M10 | Loss of C_12_H_20_O_10_ | C_15_H_10_O_6_ | 285.0395 | -3.4 | 14.54 | 71.6 |
|  |  |  | M11 | Loss of O and C_6_H_10_O_6_+Hydrogenation | C_21_H_22_O_9_ | 417.1177 | -3.5 | 13.54 | 71.3 |
| **Iridoids** | P30 | Geniposide | M12 | Loss of C_7_H_12_O_8_+Demethylation | C_10_H_12_O_4_ | 195.0661 | -1.1 | 12.29 | 76.7 |
|  |  |  | M13 | Loss of C_7_H_12_O_7_+Methylation | C_12_H_16_O_5_ | 239.0923 | -0.7 | 12.59 | 75.0 |
|  |  |  | M14 | Loss of CH_2_O_3_ and C_2_H_4_O_3_ | C_15_H_20_O_6_ | 295.1177 | -3.6 | 14.19 | 77.9 |
|  |  |  | M15 | Loss of CH_2_O_3_ and C_2_H_4_O_3_ | C_15_H_20_O_6_ | 295.1178 | -3.2 | 13.17 | 75.2 |
|  |  |  | M16 | Loss of C_7_H_12_O_7_+Sulfate Conjugation | C_11_H_14_O_8_S | 305.0334 | -0.8 | 10.11 | 61.8 |
|  |  |  | M17 | Loss of CH_2_O_3_ and O+Loss of Hydroxymethylene | C_16_H_22_O_7_ | 325.1285 | -2.3 | 13.60 | 75.1 |
|  |  |  | M18 | Loss of CH_2_O_3_ and O+Loss of Hydroxymethylene | C_16_H_22_O_7_ | 325.1285 | -2.5 | 14.06 | 76.8 |
|  |  |  | M19 | Loss of CH_2_O_3_+Loss of Hydroxymethylene | C_16_H_22_O_8_ | 341.1225 | -4.8 | 14.18 | 74.6 |
|  |  |  | M20 | Loss of CH_2_O_3_+Loss of Hydroxymethylene | C_16_H_22_O_8_ | 341.1228 | -4 | 14.38 | 73.1 |
|  |  |  | M21 | Loss of C_2_H_4_O_3_ | C_16_H_22_O_9_ | 357.1173 | -5.1 | 10.37 | 69.4 |
|  |  |  | M22 | Loss of C_2_H_4_O_3_ | C_16_H_22_O_9_ | 357.1183 | -2.3 | 11.41 | 77.9 |
|  |  |  | M23 | Loss of C_2_H_4_O_3_+Hydrogenation | C_16_H_24_O_9_ | 359.1336 | -3.3 | 10.79 | 72.1 |
|  |  |  | M24 | Loss of C_2_H_4_O_3_+Hydrogenation | C_16_H_24_O_9_ | 359.1338 | -2.6 | 8.98 | 74.0 |
|  |  |  | M25 | Loss of C_2_H_4_O_2_ | C_16_H_22_O_10_ | 373.112 | -5.4 | 10.64 | 69.4 |
|  |  |  | M26 | Loss of CH_2_O_3_+Demethylation to Carboxylic Acid | C_17_H_22_O_11_ | 401.1077 | -3.1 | 12.79 | 77.7 |
|  | P20 | Genipin 1-gentiobioside | P30 | Glucoside hydrolysis | C_17_H_24_O_10_ | 387.1296 | -0.2 | 10.17 | 81.2 |
|  |  |  | M26 | Glucoside hydrolysis+Demethylation to Carboxylic Acid | C_17_H_22_O_11_ | 401.1077 | -3.1 | 12.79 | 77.7 |
|  |  |  | M25 | Glucoside hydrolysis and Loss of CH_2_ | C_16_H_22_O_10_ | 373.112 | -5.4 | 10.64 | 69.4 |
|  |  |  | M16 | Glucoside hydrolysis +Sulfate Conjugation | C_11_H_14_O_8_S | 305.0334 | -0.8 | 10.11 | 61.8 |
| **Glycosides (Monoterpenoids)** | P14 | Jasminoside B/F | M8 | Loss of C_10_H_14_O_3_+Demethylation to Carboxylic Acid | C_6_H_10_O_7_ | 193.0351 | -1.3 | 14.77 | 75.0 |
|  |  |  | M23 | Ketone Formation | C_16_H_24_O_9_ | 359.1336 | -3.3 | 10.79 | 72.6 |
|  |  |  | M24 | Ketone Formation | C_16_H_24_O_9_ | 359.1338 | -2.6 | 8.98 | 75.4 |
|  |  |  | M27 | Loss of C_6_H_10_O_6_+Methylation | C_11_H_18_O_2_ | 181.1234 | 0 | 14.65 | 75.0 |
|  |  |  | M28 | Loss of C_6_H_10_O_5_ | C_10_H_16_O_3_ | 183.1027 | 0.3 | 9.48 | 80.2 |
|  |  |  | M29 | Loss of C_6_H_10_O_5_+Oxidation | C_10_H_16_O_4_ | 199.0977 | 0.8 | 8.72 | 76.3 |
|  |  |  | M30 | Loss of C_6_H_10_O_5_+Demethylation to Carboxylic Acid | C_10_H_14_O_5_ | 213.0769 | 0.3 | 7.71 | 76.2 |
|  |  |  | M31 | Loss of O and O+Loss of Hydroxymethylene | C_15_H_24_O_5_ | 283.1538 | -4.6 | 14.99 | 49.1 |
|  |  |  | M32 | Loss of O and O+Loss of Hydroxymethylene | C_15_H_24_O_5_ | 283.1543 | -3 | 15.17 | 53.0 |
|  |  |  | M33 | Loss of O and O+Loss of Hydroxymethylene | C_15_H_24_O_5_ | 283.1543 | -3 | 15.46 | 48.2 |
|  |  |  | M34 | Loss of O+Loss of Hydroxymethylene | C_15_H_24_O_6_ | 299.149 | -3.5 | 12.71 | 74.2 |
|  |  |  | M35 | Loss of O and O+Hydrogenation | C_16_H_28_O_6_ | 315.18 | -4.1 | 12.17 | 73.6 |
|  |  |  | M36 | Loss of O and O+Hydrogenation | C_16_H_28_O_6_ | 315.1806 | -2.2 | 11.55 | 75.2 |
|  |  |  | M37 | Loss of O | C_16_H_26_O_7_ | 329.1604 | -0.5 | 9.07 | 78.6 |
|  |  |  | M38 | Loss of O+Hydrogenation | C_16_H_28_O_7_ | 331.1748 | -4.5 | 10.42 | 68.9 |
|  |  |  | M39 | Loss of O+Hydrogenation | C_16_H_28_O_7_ | 331.1749 | -4 | 10.82 | 70.9 |
|  |  |  | M40 | Loss of O+Hydrogenation | C_16_H_28_O_7_ | 331.1755 | -2.1 | 10.27 | 75.3 |
|  |  |  | M41 | Oxidation | C_16_H_26_O_9_ | 361.1493 | -3.1 | 11.14 | 73.4 |
|  |  |  | M42 | Phosphorylation | C_16_H_27_O_11_P | 425.1249 | 7.3 | 12.73 | 63.4 |
|  |  |  | M43 | Desaturation | C_16_H_24_O_8_ | 343.1385 | -3.8 | 11.01 | 74.6 |
|  |  |  | M44 | Desaturation | C_16_H_24_O_8_ | 343.1388 | -2.9 | 14.57 | 75.6 |
|  |  |  | P44 | Loss of C_6_H_10_O_5_ | C_10_H_16_O_3_ | 183.1027 | 0.3 | 11.58 | 80.2 |
| **Organic acids** | P65 | 3-Isobutylglutaric acid | M45 | Desaturation | C_9_H_14_O_4_ | 185.0822 | 1.6 | 8.03 | 75.6 |
|  | P36 | 3-O-Feruloylquinic acid | M46 | Loss of C_7_H_10_O_5_ | C_10_H_10_O_4_ | 193.0512 | 3 | 9.92 | 73.2 |
|  |  |  | M47 | Loss of CH_2_ and C_7_H_10_O_6_+Sulfate Conjugation | C_9_H_8_O_6_S | 242.9986 | 6.9 | 9.82 | 38.9 |
|  |  |  | M48 | Loss of CH_2_ and C_7_H_10_O_5_+Sulfate Conjugation | C_9_H_8_O_7_S | 258.9919 | 0.2 | 9.76 | 58.7 |
|  |  |  | M49 | Loss of CH_2_ and C_7_H_10_O_5_+Oxidation | C_9_H_8_O_5_ | 195.0306 | 3.4 | 12.30 | 71.5 |
|  | P70 | 4-Sinapoyl-5-caffeoylquinic acid | M46 | Loss of CH_2_ and C_16_H_16_O_9_ | C_10_H_10_O_4_ | 193.0512 | 3 | 9.92 | 72.5 |
|  |  |  | M47 | Loss of C_18_H_20_O_10_+Sulfate Conjugation | C_9_H_8_O_6_S | 242.9986 | 6.9 | 9.82 | 37.7 |
|  |  |  | M48 | Loss of C_18_H_20_O_9_+Sulfate Conjugation | C_9_H_8_O_7_S | 258.9919 | 0.2 | 9.76 | 50.0 |
|  |  |  | M50 | Loss of C_18_H_20_O_10_+Glycine Conjugation | C_11_H_11_NO_4_ | 220.0614 | -0.8 | 10.84 | 75.0 |
|  |  |  | M51 | Loss of C_16_H_16_O_9_+Methylation | C_12_H_14_O_4_ | 221.0816 | -1.6 | 14.23 | 75.0 |
|  |  |  | M52 | Loss of C_16_H_16_O_8_+Sulfate Conjugation | C_11_H_12_O_8_S | 303.0183 | 0.8 | 9.56 | 50.0 |
|  |  |  | M53 | Loss of C_11_H_10_O_5_+Hydrogenation | C_16_H_20_O_8_ | 339.1069 | -4.7 | 13.93 | 68.2 |
|  |  |  | M54 | Loss of C_11_H_10_O_5_+Hydrogenation | C_16_H_20_O_8_ | 339.1069 | -4.7 | 14.09 | 68.2 |
|  |  |  | M55 | Loss of CH_2_ and CH_2_+Sulfate Conjugation | C_25_H_24_O_16_S | 611.0708 | -0.6 | 10.11 | 50.0 |
|  |  |  | P36 | Loss of CH_2_ and C_9_H_6_O_4_ | C_17_H_20_O_9_ | 367.1024 | -2.9 | 10.89 | 72.9 |
| **Steroid saponins** | P98 | Dioscin | M56 | Loss of C_27_H_40_O_2_ and C_6_H_10_O_5_ | C_12_H_22_O_9_ | 311.1317 | -6.3 | 14.17 | 64.3 |
