# Supplementary material for: Chemicolome and Metabolome Profiling of Xieriga-4 Decoction, A Traditional Mongolian Medicine, Using the UPLC-QTOF/MS Approach
Source: Evid Based Complement Alternat Med. 2022 Nov 16;2022:8197364. doi: 10.1155/2022/8197364 (PMC9683986; doi:10.1155/2022/8197364)
Supplement: Supplementary Materials — Supplementary information available: Tables S1-S3 and Figures S1-S3. [file 8197364.f1.zip › Table S3 (1).docx]

Table S 3 Distribution of substance basis in vivo

| **NO.** | **Name** | **Ptoyotypes** | **Urine** | **Plasma** | **Feces** | **Brain** | **Heart** | **Kidney** | **Liver** | **Lung** | **Spleen** |
| --- | --- | --- | --- | --- | --- | --- | --- | --- | --- | --- | --- |
| 1 | Quinic acid | P1 | √ | √ | √ | - | - | - | - | - | - |
| 2 | Candicine | P3 | - | - | √ | - | - | - | - | - | - |
| 3 | Gardoside | P4 | √ | - | - | - | - | - | - | - | - |
| 4 | Geniposidic acid | P6 | √ | - | - | - | - | - | - | - | - |
| 5 | Shazhiside or its isomer | P8 | √ | - | - | - | - | - | - | - | - |
| 6 | Gentisic acid | P10 | - | - | √ | - | - | - | - | - | - |
| 7 | Feretoside | P12 | √ | - | - | - | - | - | - | - | - |
| 8 | Jasminoside D/G | P16 | √ | - | - | - | - | - | - | - | - |
| 9 | Genipin 1-gentiobioside | P20 | √ | √ | - | - | - | - | - | - | - |
| 10 | Clausenamide | P21 | √ | - | √ | - | - | - | - | - | - |
| 11 | 5-O-Feruloylquinic acid | P23 | √ | - | - | - | - | - | - | - | - |
| 12 | Phellodendrine oxide | P26 | √ | - | √ | - | - | - | - | - | - |
| 13 | N-Methylhigenamine 7-glucopyranoside | P27 | √ | - | - | - | - | - | - | - | - |
| 14 | Tetrahydrojatrorrhizine | P28 | √ | - | √ | - | - | - | - | - | - |
| 15 | Caffeic acid | P29 | √ | - | √ | - | - | - | - | - | - |
| 16 | Geniposide | P30 | √ | √ | - | - | - | - | - | - | - |
| 17 | Tembetarine | P31 | - | - | √ | - | - | - | - | - | - |
| 18 | Phellodendrine | P33 | √ | - | √ | - | - | - | - | - | - |
| 19 | Picrocrocin | P34 | √ | - | - | - | - | - | - | - | - |
| 20 | 3-O-Feruloylquinic acid | P36 | √ | - | - | - | - | - | - | - | - |
| 21 | Lotusine | P38 | - | - | √ | - | - | - | - | - | - |
| 22 | 4-O-Feruloylquinic acid | P41 | √ | - | - | - | - | - | - | - | - |
| 23 | Jasminodiol | P44 | √ | - | √ | - | - | - | - | - | - |
| 24 | Menisperine | P45 | - | - | √ | - | - | - | - | - | - |
| 25 | Ferulic acid | P48 | √ | √ | √ | - | - | - | - | - | - |
| 26 | Demethyleneberberine | P51 | √ | - | √ | - | - | - | - | - | - |
| 27 | Oxyberberine | P58 | - | - | √ | - | - | - | - | - | - |
| 28 | Berberrubine | P66 | √ | - | √ | - | - | - | √ | - | - |
| 29 | Columbamine/Jatrorrhizine | P67 | - | - | √ | - | √ | √ | - | - | - |
| 30 | Palmatine | P75 | - | - | √ | - | √ | √ | - | - | - |
| 31 | Berberine | P77 | - | √ | √ | √ | √ | √ | √ | √ | √ |
| 32 | Rutaevin | P85 | √ | - | - | - | - | - | - | - | - |
| 33 | Curcumin | P100 | √ | - | - | - | - | - | - | - | - |
| **Count** | | | **24** | **5** | **19** | **1** | **3** | **3** | **2** | **1** | **1** |
| 34 | M1 | | √ | - | - | - | - | - | √ | - | - |
| 35 | M2 | | √ | - | - | - | - | - | - | - | - |
| 36 | M3 | | √ | - | - | - | - | - | - | - | - |
| 37 | M4 | | √ | - | √ | - | - | - | - | - | - |
| 38 | M5 | | √ | - | - | - | - | - | - | - | - |
| 39 | M6 | | √ | - | - | - | - | - | - | - | - |
| 40 | M7 | | √ | - | - | - | - | - | - | - | - |
| 41 | M8 | | √ | - | - | - | - | - | - | - | - |
| 42 | M9 | | √ | - | - | - | - | - | - | - | - |
| 43 | M10 | |  | - | √ | - | - | - | - | - | - |
| 44 | M11 | | √ | - | - | - | - | - | - | - | - |
| 45 | M12 | | √ | - | - | - | - | - | - | - | - |
| 46 | M13 | | √ | - | - | - | - | - | - | - | - |
| 47 | M14 | | √ | - | - | - | - | - | - | - | - |
| 48 | M15 | | √ | - | - | - | - | - | - | - | - |
| 49 | M16 | | √ | √ | - | - | - | - | - | √ | - |
| 50 | M17 | | √ | - | - | - | - | - | √ | - | - |
| 51 | M18 | | √ | - | - | - | - | - | - | - | - |
| 52 | M19 | | √ | - | - | - | - | - | - | - | - |
| 53 | M20 | | √ | - | - | - | - | - | - | - | - |
| 54 | M21 | | √ | - | - | - | - | - | - | - | - |
| 55 | M22 | | √ | - | - | - | - | - | - | - | - |
| 56 | M23 | | √ | - | - | - | - | - | - | - | - |
| 57 | M24 | | √ | - | - | - | - | - | - | - | - |
| 58 | M25 | | √ | - | - | - | - | - | - | - | - |
| 59 | M26 | | √ | - | - | - | - | - | - | - | - |
| 60 | M27 | | √ | - | - | - | - | - | - | - | - |
| 61 | M28 | | √ | - | √ | - | - | - | - | - | - |
| 62 | M29 | | √ | - | - | - | - | - | - | - | - |
| 63 | M30 | | √ | - | - | - | - | - | - | - | - |
| 64 | M31 | | √ | - | √ | - | - | - | - | - | - |
| 65 | M32 | | √ | - | √ | - | - | - | - | - | - |
| 66 | M33 | | √ | - | √ | - | - | - | - | - | - |
| 67 | M34 | | √ | - | - | - | - | - | - | - | - |
| 68 | M35 | | √ | - | - | - | - | - | - | - | - |
| 69 | M36 | | √ | - | - | - | - | - | - | - | - |
| 70 | M37 | | √ | - | - | - | - | - | - | - | - |
| 71 | M38 | | √ | - | - | - | - | - | - | - | - |
| 72 | M39 | | √ | - | - | - | - | - | - | - | - |
| 73 | M40 | | √ | - | - | - | - | - | - | - | - |
| 74 | M41 | | √ | - | - | - | - | - | - | - | - |
| 75 | M42 | | √ | - | - | - | - | - | - | - | - |
| 76 | M43 | | √ | - | - | - | - | - | √ | - | - |
| 77 | M44 | | √ | - | - | - | - | - | - | - | - |
| 78 | M45 | | √ | - | - | - | - | - | - | - | - |
| 79 | M46 | | √ | - | - | - | - | - | - | - | - |
| 80 | M47 | | √ | √ | - | - | - | - | - | - | - |
| 81 | M48 | | √ | √ | - | - | - | - | - | - | - |
| 82 | M49 | |  | - | √ | - | - | - | - | - | - |
| 83 | M50 | | √ | - | - | - | - | - | - | - | - |
| 84 | M51 | | √ | - | - | - | - | - | - | - | - |
| 85 | M52 | | √ | - | - | - | - | - | - | - | - |
| 86 | M53 | | √ | √ | - | - | - | - | - | - | - |
| 87 | M54 | | √ | - | - | - | - | - | - | - | - |
| 88 | M55 | | √ | - | - | - | - | - | - | - | - |
| 89 | M56 | | √ | - | - | - | - | - | - | - | - |
| **Count** | | | **54** | **4** | **7** | **0** | **0** | **0** | **3** | **1** | **0** |
| **All** | | | **78** | **9** | **26** | **1** | **3** | **3** | **5** | **2** | **1** |

"√" indicates detection
